# Changes of lake organic carbon sinks from closed basins since the Last Glacial Maximum and quantitative evaluation of human impacts

**DOI:** 10.1186/s13021-021-00191-6

**Published:** 2021-09-17

**Authors:** Yu Li, Xinzhong Zhang, Lingmei Xu, Yuxin Zhang, Wangting Ye, Yichan Li

**Affiliations:** 1 Key Laboratory of Western China’s Environmental Systems (Ministry of Education), College of Earth and Environmental Sciences, Center for Hydrologic Cycle and Water Resources in Arid Region, Lanzhou University, Lanzhou, China; 2 Department of Geosciences and Geography, University of Helsinki, Helsinki, Finland; 3 Department of Geosciences, University of Arkansas, Fayetteville, USA

**Keywords:** Carbon sinks, Closed basins, Human activities, Last Glacial Maximum, Climate change

## Abstract

**Background:**

Closed basins occupy 21% of the world’s land area and can substantially affect global carbon budgets. Conventional understanding suggests that the terminal areas of closed basins collect water and carbon from throughout the entire basin, and changes in lake organic carbon sinks are indicative of basin-wide organic carbon storages. However, this hypothesis lacks regional and global validation. Here, we first validate the depositional process of organic carbon in a typical closed-basin region of northwest China using organic geochemical proxies of both soil and lake sediments. Then we estimate the organic carbon sinks and human impacts in extant closed-basin lakes since the Last Glacial Maximum (LGM).

**Results:**

Results show that 80.56 Pg organic carbon is stored in extant closed-basin lakes mainly found in the northern mid-latitudes. Carbon accumulation rates vary from 17.54 g C m^−2^ yr^−1^ during modern times, 6.36 g C m^−2^ yr^−1^ during the mid-Holocene and 2.25 g C m^−2^ yr^−1^ during the LGM. Then, we evaluated the influence by human activities during the late Holocene (in the past three thousand years). The ratio of human impacts on lake organic carbon storage in above closed basins is estimated to be 22.79%, and human-induced soil organic carbon emissions in the past three thousand years amounted to 207 Pg.

**Conclusions:**

While the magnitude of carbon storage is not comparable to those in peatland, vegetation and soil, lake organic carbon sinks from closed basins are significant to long-term terrestrial carbon budget and contain information of climate change and human impact from the whole basins. These observations improve our understanding of carbon sinks in closed basins at various time scales, and provide a basis for the future mitigation policies to global climate change.

**Supplementary Information:**

The online version contains supplementary material available at 10.1186/s13021-021-00191-6.

## Background

**Fig. 1:**
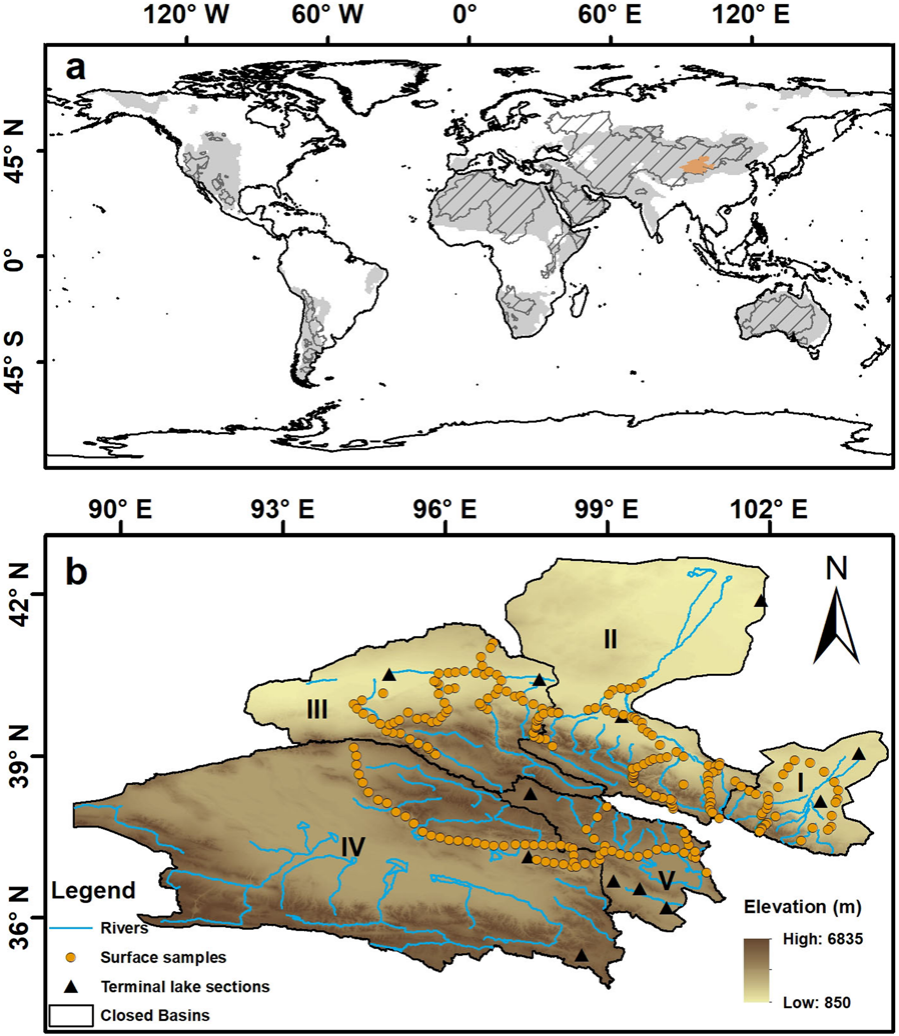
Overview maps of study regions. **a** Global map of closed basins and locations of closed basins of the Qilian Mountains. The shaded parts show global drylands where aridity indexes are lower than 0.65 and the gray bars indicate global closed basins where there are no outflows to the oceans. **b** Detailed map of closed basins of the Qilian Mountains and locations of sampling sites. The Roman numerals represent various closed drainage basins, I: Shiyang River drainage basin, II: Heihe River drainage basin, III: Shule River drainage basin, IV: Qaidam drainage basin; V: Qinghai Lake drainage basin

Closed basins, defined as the regions where surface flow is unable to break topographic barriers and thus retains in a landlocked storage [32], occupy 30,873,561 km^2^ or about 21% of the world’s land area [26, 53]. Largely due to far distances from oceans, most closed basins are located in drylands where aridity indexes are lower than 0.65 [19],Fig. 1a). Closed-basin carbon sinks have recently been recognized as significant components of the terrestrial carbon budgets and are believed to play important roles in the global carbon cycle [1, 25, 26, 53]. It has been estimated that closed basins currently bury at least 0.152 Pg of dissolved inorganic carbon per year, which is comparable to the deep ocean carbon burial of 0.2 Pg C yr^−1^ [26, 40]. Total dissolved inorganic carbon pool during the mid-Holocene was up to 739.1 Pg C in global closed basins, higher than contemporaneous carbon stock of global peatland (305 Pg C) [26, 50]. However, controversies still exist in the mechanism and actual magnitude of organic carbon sink in closed basins.

As a unique geographic unit, closed basins usually have a relatively isolated and integrated carbon cycle [25, 26, 58]. Since there is no outlet, terminal lake can be considered as the “ocean” for closed basin. The terminal lake concentrates sedimentary materials from throughout the whole basin, having the potential to sequester extensive terrestrial organic matter [26, 51]. This characteristic makes organic carbon storage in terminal lakes an advantageous indicator to imply carbon change of the whole basins. In addition, many studies have demonstrated that the recent variations in lacustrine deposits are closely related to anthropogenic factors [3, 16, 21]. Therefore, the terminal lake carbon sink can also be used to evaluate the human impacts in closed basins.

Closed basins of the Qilian Mountains, located in the arid region of northwest China, are appropriate areas to explore carbon storage mechanism (Fig. 1b). Here we evaluate changes of lake organic carbon sinks from closed basins of the Qilian Mountains as a case study for research into the mechanisms of closed-basin carbon sink. 234 soil samples were collected in total for analysis of organic proxies in order to validate the transformation of soil organic carbon in closed basins of the Qilian Mountains. The organic carbon content and ^14^C/OSL (optically stimulated luminescence) dating results from twelve lake records in this region were used to estimate organic carbon changes since the LGM and human impacts during the late Holocene (Additional file 1: Table S1 and S2). Then we evaluated organic carbon sinks in extant closed-basin lakes at a larger spatial scale, based on the knowledge of carbon cycle in closed basins of the Qilian Mountains. The pH, alkalinity and salinity derived from 82 closed-basin lakes were used to define weight coefficients for three different lake types (carbonate, sulfate and chlorine). Organic carbon accumulation rates since the LGM derived from 39 closed-basin lakes were reassigned according to the weight coefficients. Finally, we calculated the organic carbon storage in closed-basin lakes (mainly located in northern mid-latitudes) since the LGM and evaluated the human impacts both on organic carbon sinks and on soil organic carbon emissions.

## Results and discussion

### Lake organic carbon storage in closed basins of the Qilian Mountains

**Fig. 2:**
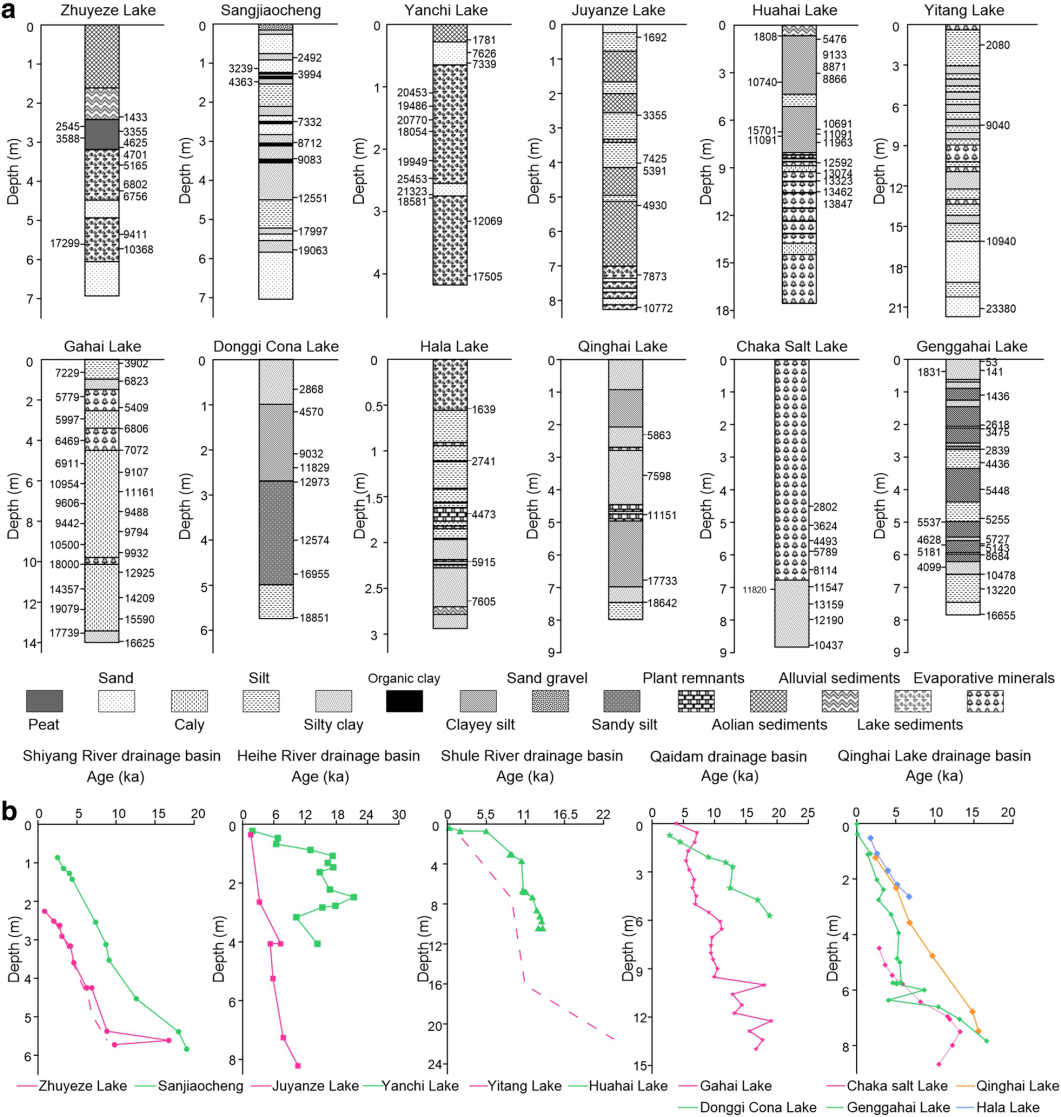
The lithology and ^14^C/OSL dating results from 12 terminal lakes in the closed basins of the Qilian Mountains. **a** Lithology of the 12 lake sections. **b** Comparison of the calibrated ^14^C (solid lines) and OSL ages (dashed lines) in the 5 closed basins of the Qilian Mountains. (Lithology data of Qinghai Lake are from Liu et al. [28], other lithology and ^14^C/OSL data sources see Additional file 1: Table S1)

Accurate chronologies are of crucial importance in long-term carbon sequestration studies, however, ^14^C ages in arid areas are often affected by the carbon reservoir which makes the measured age older than it should be [29, 36, 43]. There are two common approaches to solve this problem in lake sediments: dating different materials (such as terrestrial plant remains, shells, charcoals and bulk organic matters) by the same dating method and dating the same material by different dating methods (such as ^14^C, OSL, Electron Spin Resonance and U-series dating). In this study, ^14^C ages were first corrected to remove any possible old-carbon effect according to age differences based on various dating materials or methods in original publications, then the corrected ^14^C ages were calibrated to calendar years (Cal BP) using the Calib 7.1 program (Additional file 1: Table S1) [48]. Based on the dating results in closed basins of the Qilian Mountains, especially the consistence between OSL and calibrated ^14^C ages in Zhuyeze Lake, we believe that the reservoir effect is relatively small in this region, which is acceptable for analyzing the millennial-scale carbon sink changes (Fig. 2b) [30].

**Fig. 3:**
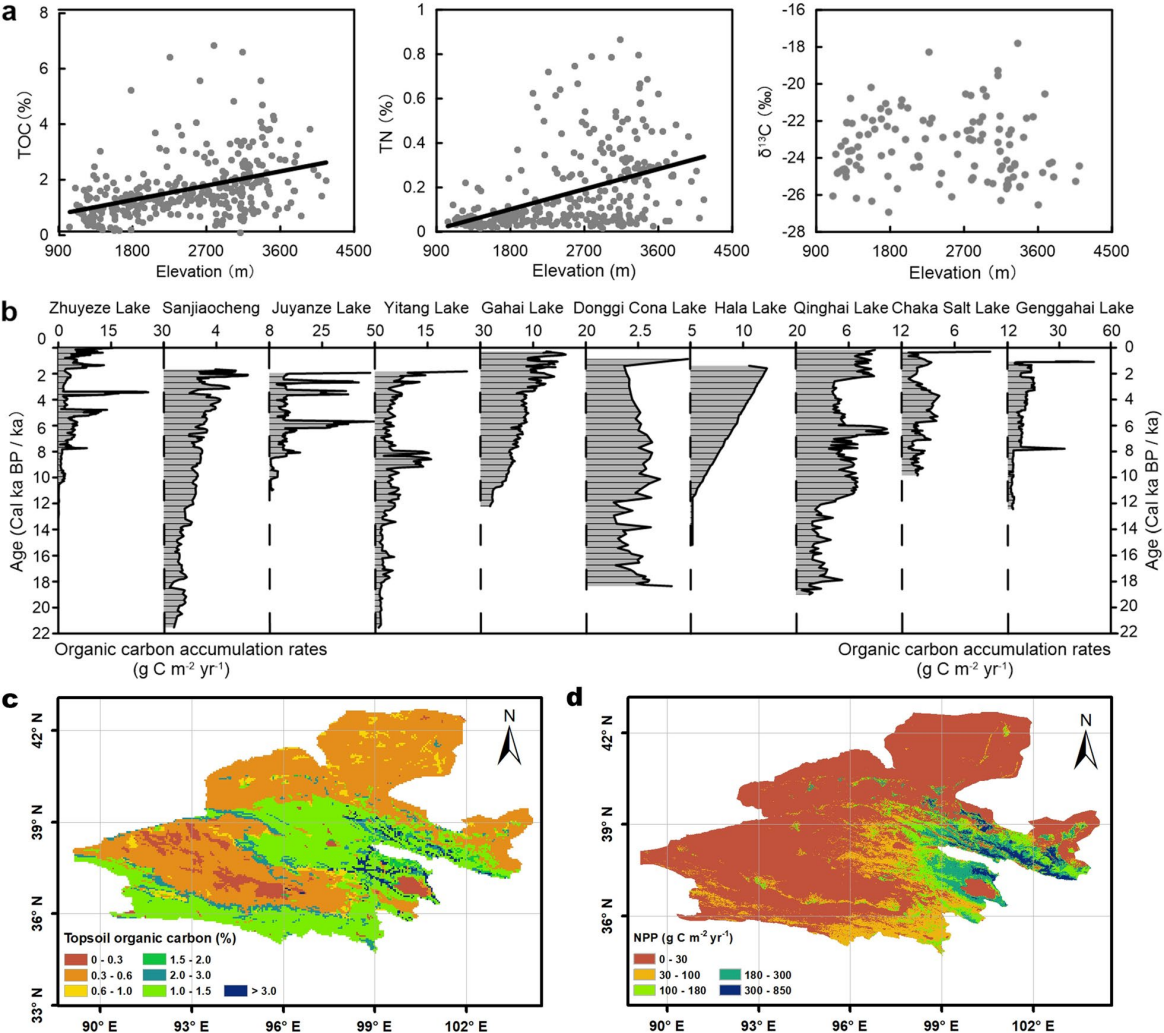
Organic carbon related data from lake records and modern observations in closed basins of the Qilian Mountains. **a** TOC, TN, organic δ^13^C from soil samples versus elevations. **b** Organic carbon accumulation rates in closed-basin lakes of the Qilian Mountains. **c** Topsoil organic carbon contents (0–30 cm) [56]. **d** Modern NPP distributions (year 2000–2015) [45]

Conventional understanding of closed basins suggests their terminal areas collect water and carbon from throughout their entire drainage basins [26, 53]. Here we validated this hypothesis in closed basins of the Qilian Mountains using organic geochemical proxies of both soils and lake sediments. Total Organic Carbon (TOC) contents from soil samples across closed basins of the Qilian Mountains show an overall increasing trend along with rising elevations, and this changing trend is in accordance with those in the topsoil organic carbon content and modern net primary production (NPP) distributions which show that higher elevations have higher TOC and NPP (Fig. 3a, c, d). It is thus illustrated that the samples are representative and can effectively indicate the soil organic carbon changes of this region. Soil organic δ^13^C are generally scattered between − 26.96 ‰ to − 17.83 ‰ with an average value of − 23.41 ‰, indicating C_3_ plants with light δ^13^C values dominate the terrestrial plant types (Fig. 3a). This is consistent with the results from previous paleovegetation studies that concluded C_4_ plants have not developed in the arid area of northwest China since the LGM [8, 59]. Therefore, the relatively light organic δ^13^C in lake records from closed basins of the Qilian Mountains is likely related to organic carbon inputs from terrestrial C_3_ plants rather than C_4_ plants.

The long-term variations of lithology and organic geochemical proxies in lake sediments follow a progressive depositional sequence (Fig. 2 and 3, Additional file 1: Fig. S2). During the LGM and following deglacial period, most lake records show relatively low TOC and various organic carbon sources. For example, fluvial facies and high organic δ^13^C in Sanjiaocheng section indicate a large proportion of organic carbon input from terrestrial C_3_ plants, whereas autochthonous organic carbon dominate in Lakes Qinghai and Yitang indicated by low C/N and δ^13^C [47, 59, 60]. During the Holocene, the relatively high C/N and TOC values in overlying lacustrine sections of most lakes suggest a stronger mixing effect of both terrestrial and aquatic sources of organic carbon, namely higher proportions of terrestrial organic carbon input than those during the LGM. Whatever the sources of organic carbon are, TOC is typically higher during the Holocene than during the LGM, implying similar responses of both terrestrial and aquatic ecosystems to long-term climate change. Changes of organic carbon sinks in the terminal lakes of closed basins can therefore indicate not only lake primary productivity but also terrestrial ecosystem productivity in their catchments.

Finally, the mean carbon accumulation rate in the 12 closed-basin lakes of the Qilian Mountains is estimated to 10.74 g C m^−2^ yr^−1^ in modern times, 6.31 g C m^−2^ yr^−1^ in the mid-Holocene and 1.76 g C m^−2^ yr^−1^ in the LGM (Additional file 1: Table S3). Using lake area calculated from HydroSHEDS products [24], lake organic carbon storage in closed basins of the Qilian Mountains since the LGM is up to 0.90 Pg C. Comparatively, the modern soil organic carbon storage amounts to 3.32 Pg C based on Regridded Harmonized World Soil Database (HWSD) v1.2 [56].

### Organic carbon sinks and human impacts in extant closed-basin lakes

**Fig. 4:**
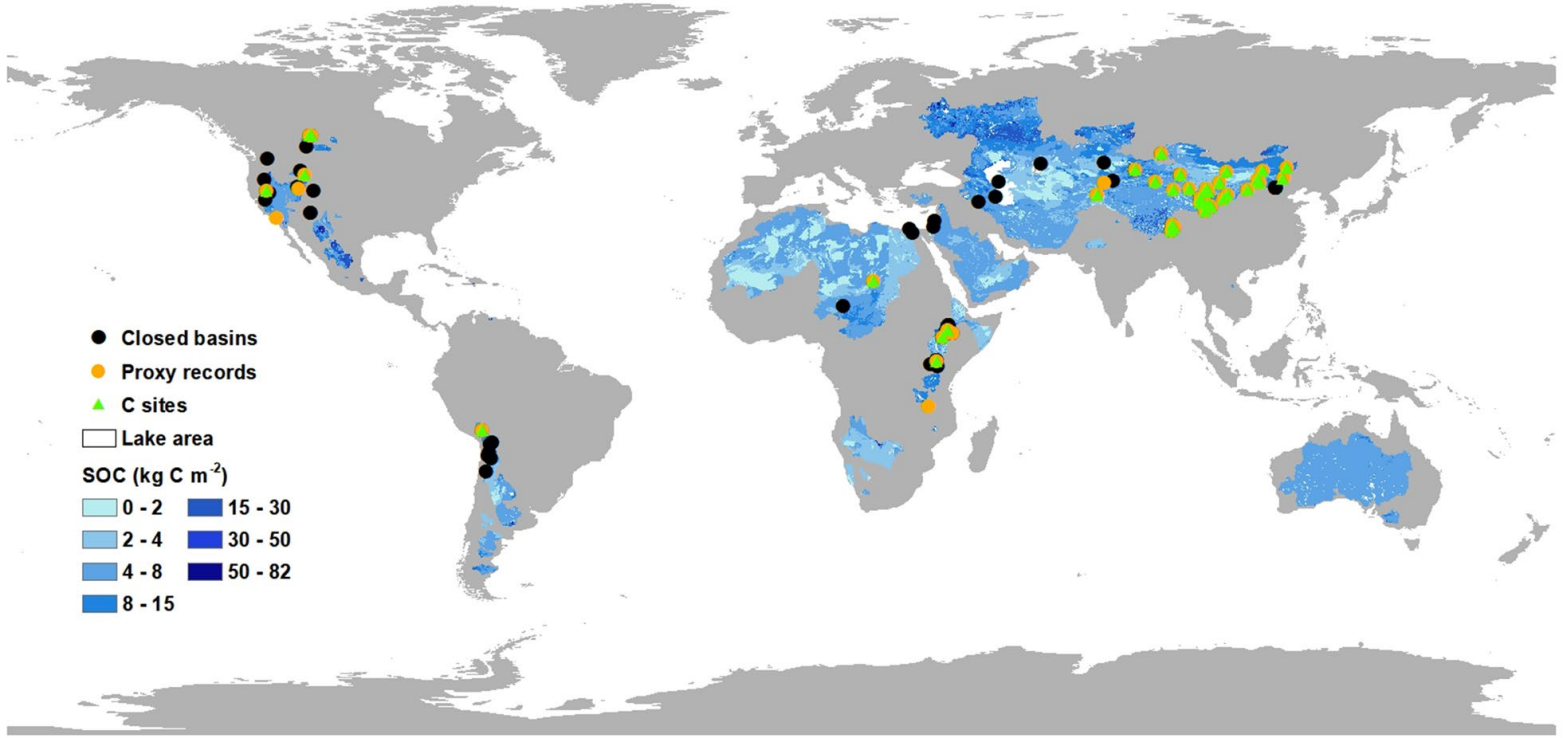
Soil organic carbon density in contemporary closed basins and site locations of lake records used in this study. Black dots show the 82 closed-basin lakes with available geophysical-chemical data of lake water. Yellow dots show the 43 closed-basin lakes with available organic geochemical proxies. Green triangles show the 39 closed-basin lakes with available organic carbon data

Following the analysis of closed basins of the Qilian Mountains, we validate and evaluate the long-term organic carbon changes in other closed, mainly northern mid-latitude, basins with available organic geochemical proxies from their terminal lakes (Fig. 4, Additional file 1: Table S4, Figs. S4 and S5). During the LGM, Lakes Karakul, Luobupo and Zabuye in Asia, Lakes Bear and Red Rock in North America all show relatively low TOC, indicating similar change trends to those in closed basins of the Qilian Mountains (Additional file 1: Fig. S3) [12, 18, 31, 37, 55]. During the Holocene, comparatively high TOC and C/N in most lake records imply higher productions in both terrestrial and aquatic ecosystems. According to the available chronologies from closed-basin lakes, most records show relatively weak carbon reservoir effect generally within 1000–2000 years which are not considered too significant for a millennial-scale research after using the carbon reservoir correction from original publications [9, 12, 17, 37]. Therefore, the similar carbon sink mechanisms indicate that the experiences from closed basins of the Qilian Mountains can be used to assess organic carbon storages in other closed basins.

Uneven spatial distributions and research focuses in closed-basin lakes pose limits on available organic carbon data, especially in Africa and Australia. In order to more reasonably evaluate the carbon sinks at a larger spatial scale under a limited data condition, we firstly classified the extant closed-basin lakes into three types (carbonate, sulfate and chlorine), according to the geophysical-chemical characteristics of lake water (Additional file 1: Table S5) [26]. Terminal lake water characteristics can be considered as the comprehensive function of multiple regional factors and thus have great influence on organic carbon storage [46]. The weight coefficients were calculated as 0.021, 0.221 and 0.757 for carbonate, sulfate and chlorine lake types, respectively (see Methods for details). Then, the organic carbon accumulation rates since the LGM were multiplied by the weight coefficients according to their lake types. Results show modern mean organic carbon accumulation rate (CAR) is 17.54 g C m^−2^ yr^−1^, 6.36 g C m^−2^ yr^−1^ in the mid-Holocene and 2.25 g C m^−2^ yr^−1^ in the LGM (Additional file 1: Table S6). The total organic carbon sink in extant closed-basin lakes is 80.56 Pg C since the LGM. It is worth noting that the lake organic carbon storages calculated in this study is based on modern extant lake area (about 735,492.2 km^2^ [24]), and thus this estimate only represents the final residue of lake organic carbon sinks. However, the use of consistent lake area in the long-term calculation gives an advantage in quantitative evaluation of human impacts.

During the last few thousand years, human-induced land-use change directly affected the catchment landscape and regional hydrological response, and thus had strong influences on organic carbon storages both in soils and lakes [57]. Human-induced deforestation, pastoralism and agricultural cultivation destroy the stability of regional terrestrial ecosystems, resulting in accelerated soil erosion and lake sediment accumulation [10, 54]. According to recent authoritative studies regarding anthropogenic transformation of environments [3, 16], this paper proposes that intense human interference around the world begins three thousand years ago, and this point is indeed supported by higher CAR but relatively low TOC during the late Holocene in closed-basin lakes (Additional file 1: Table S7). This unusual phenomenon is closely related to terrestrial organic matter input due to human induced land-use change [54]. Therefore, we assumed the carbon accumulation from 22 to 3 ka is not affected by human activities and completely represents natural variations, while the carbon accumulation during the last 3 ka suggests information on both natural and anthropogenic factors. According to curve fitting analysis of organic carbon accumulation rate from 22 to 3 ka, we obtained the potential natural CAR after 3 ka excluding human impacts. According to Eq. (8) in Methods, the ratio of human impacts is estimated to 22.79% on the organic carbon sinks in extant closed-basin lakes during the past three thousand years. However, the organic carbon sinks in terminal lakes contain only a portion of destroyed soil organic carbon, a majority of it being oxidized and released into the atmosphere. According to Eq. (9), we further estimate human-induced soil organic carbon emissions in the past three thousand years, amounting to 207 Pg C.

### Role of closed-basin organic carbon sinks in global carbon budgets

**Fig. 5:**
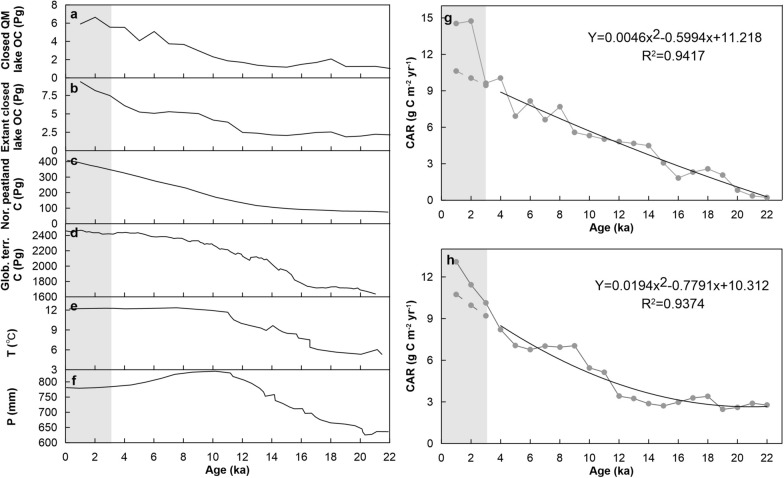
Comparisons of organic carbon storage in extant closed-basin lakes with other terrestrial carbon storage and climate background since the LGM. **a** Organic carbon storage in closed-basin lakes of the Qilian Mountains and **b** in extant closed-basin lakes. **c** Northern peatland carbon stock [50]. **d** Global terrestrial carbon storage [22]. **e** Global land temperature change and **f** precipitation change [50]. **g** Temporal variation patterns of organic carbon accumulation rate at 1000-year bins in closed-basin lakes of the Qilian Mountains and **h** in extant closed-basin lakes. The gray shadow denotes the period including significant human impacts. The gray dashed line indicates processed CAR values when human impacts have been excluded

Regional process/mechanism studies help resolve the controversies regarding the organic carbon sink in closed basins and are key to achieving a comprehensive and credible global carbon budget [26]. By translating the results of our analysis of long-term carbon cycle mechanisms to other closed basins outside the Qilian Mountains, we provide a global perspective of organic carbon storage in closed-basin lakes. The organic carbon sink in terminal lakes indicates an overall increasing trend from LGM to present, in agreement with previous estimates of increases in northern peatland and global terrestrial carbon storage (Fig. 5) [22, 50]. Comparing the LGM to the Holocene, it seems that extant closed-basin lakes around the globe have lower organic carbon content and storage during glacial period than during interglacials. However, the timing of peak sedimentation and therefore organic carbon burial would have varied widely among different closed basins, due to regional differences in climate, vegetation and lake evolution since the LGM. Pluvial Great Basin during the LGM and Green Sahara during the Holocene are prime examples of this difference in lake evolution [14, 27, 42, 44]. Another source of uncertainty in lake organic carbon sink estimation is that many closed-basin lakes, active since the LGM, have dried out in recent times, exposing organic carbon in lake sediments to oxidizing atmosphere conditions.

Based on the methods we used in this study, the total organic carbon pool in the extant closed-basin lakes from LGM to present is estimated to 80.56 Pg C. It is relatively small in magnitude compared with other estimations of carbon sinks, such as inorganic carbon sink of closed basins (739.1 Pg C in the mid-Holocene) [26], peatland carbon burial (560 Pg C since the LGM) [50], organic carbon storage in the northern permafrost region (1672 Pg C) [49], vegetation carbon storage (405 Pg C in the LGM, 771 Pg C in pre-industrial Holocene) [41], soil carbon storage (520 Pg C in the LGM, 682 Pg C in pre-industrial Holocene) [41] and terrestrial carbon storage (3640 Pg C in the LGM) [11]. However, the organic carbon storage in the terminal areas of closed basins can be considered as an effective indicator of carbon change throughout the entire basin. On this basis, we further evaluate the human-induced soil organic carbon emissions in the past three thousand years amounting to 207 Pg. This magnitude is larger compared to modern soil organic carbon storage (170.58 Pg).

Human activities have transformed and managed landscapes for thousands of years, altering global pattern of ecosystem functioning, carbon cycle, and climate [16]. Inland water carbon sinks and their influencing factors are key constituents associated with global ecosystem change, playing a potentially important role in global carbon budgets by balancing the carbon supplies from the atmosphere and associated watersheds and the net demand of primary producers [15]. Humans have deeply participated in above processes, and their impacts on both carbon storages and emissions will potentially change the global carbon cycle. Intergovernmental Panel on Climate Change (IPCC) has reported that human-induced carbon emissions contribute significantly to global warming, with net CO_2_ emissions of 39.1 ± 3.2 Pg yr^−1^ during 2007 to 2016 and global land surface air temperature increasing by 1.53℃ since the pre-industrial period [20]. As research on carbon-climate feedbacks is crucial for both political and social decision-making globally, unremitting efforts are still required to quantitatively estimate human impacts to global carbon budget [6, 26].

## Conclusion

This paper presents a regional study of organic carbon deposition in terminal lakes from the closed basins of the Qilian Mountains and a global study calculating and evaluating the lake organic carbon sinks in closed basins since the LGM. We find that the overall organic carbon sink in terminal lakes of closed basins is certainly not as significant as other carbon sinks, since its magnitude (80.56 Pg C from LGM to present) is much smaller than those in the peatland, vegetation and soil. However, it is still an important component of the terrestrial carbon budget, and the changes in lake organic carbon sinks are indicative of basin-wide organic carbon storages. On this basis, we further estimate that human-induced soil organic carbon emissions in the past three thousand years amount to 207 Pg. Our results contribute to the understanding on carbon sinks in closed basins at various time scales and provide bases for the future mitigation policies to global climate change.

## Methods

### Data collection and analysis in closed basins of the Qilian Mountains

Located at the transition area between drylands of northwest China, East Asian monsoonal region and Qinghai-Tibet Plateau, modern climate in the Qilian Mountains is strongly influenced by the combined effect of the Asian monsoons and westerly winds. Five closed basins cover this region, except for the Datong River drainage basin in the southeast part belonging to the Yellow River basin (Fig. 1b). Due to the wide range of altitude variation, the land use and land cover patterns show significant vertical zonality, with forest concentrated at high mountains and farmland distributed in lower basins (Additional file 1: Fig. S1). Since the Han Dynasty (2000 years ago), this region has been the core area of Silk Road connecting the central China to the west world, and thus affected extensively by human activities. Given the relatively isolated carbon cycle and long development history, closed basins of the Qilian Mountains are ideal for regional carbon sink and human impact researches.

In September 2018, we collected 234 surface soil samples in closed basins of the Qilian Mountains (Fig. 1b). The sampling sites (at least 5 km from each other) were taken as far away as possible from residential/industrial/agricultural areas so as to be more indicative of natural vegetation and landscapes. Due to the low accessibility in many places of this mountainous area, soil sampling was done along main roads (though at least 50 m away from said roads), resulting in some limited coverage of sampling sites, especially in the south part of the Qilian Mountains. In addition, the southern Qaidam Basin and the northern Heihe Basin are far away from the core area of Qilian Mountains we focused on, so we didn’t take soil samples from these areas. However, the sample ensembles are still fairly representative of overall organic geochemical characteristics of surface soils in closed basins of the region.

Lake sediment samples at Zhuyeze Lake in Shiyang River drainage basin were collected in October 2004 from a hand-excavated section near the edge of the lake at elevation of 1309 m. The section was sampled at 2 cm intervals in the lake sediment layers and at 5 cm intervals otherwise, resulting in 292 samples for organic geochemical analyses. Six AMS ^14^C dates and seven conventional ^14^C dates were obtained from the shells and bulk organic matter, respectively. Also, totally 83 lake sediment samples at Yanchi Lake in Heihe River drainage basin were collected from a section at 5 cm intervals excavated in the middle part of the dried lake basin. Twelve AMS ^14^C dates and two conventional ^14^C dates were obtained from the pollen concentrates and bulk organic matter, respectively.

All above samples were measured for organic geochemical proxies of total organic carbon (TOC), carbon–nitrogen ratio (C/N) and stable carbon isotope (δ^13^C) in Analysis and Testing Center of Lanzhou University, and chronology of lake sediment samples were completed for conventional ^14^C dating in Dating Laboratory of Lanzhou University and for Accelerator Mass Spectrometry (AMS) ^14^C dating in Dating Laboratory of Peking University. Data from the other 10 lake records with measured organic geochemical proxies and reliable chronologies in closed basins of the Qilian Mountains were compiled from published resources (Fig. 1b, Additional file 1: Tables S1 and S2).

Organic geochemical proxies of TOC, C/N and δ^13^C commonly used in paleoenvironmental studies were applied to validate the depositional process of organic carbon in closed basins of the Qilian Mountains. TOC can directly indicate the organic matter input, and further imply the regional primary productivity [5]. C/N is considered an indicator of organic matter origin in lacustrine sediments [52]. C/N less than 10 indicates the organic matter mainly originates from aquatic plants, whereas C/N between 14 and 23 means terrestrial organic matter input [33–35]. The organic δ^13^C can suggest the relative contribution of organic carbon from various terrestrial plants sources, with − 34‰ to − 23‰ in C_3_ plants, − 22‰ to − 6‰ in C_4_ plants and − 20‰ to − 10‰ in crassulacean acid metabolism (CAM) plants [39]. Likewise, the relative abundance of different aquatic plants during the sedimentary period can be inferred according to the ranges of δ^13^C value. Lake aquatic vegetation is dominated by submerged plant when it is -12‰ to − 20‰, emergent plant when it is − 24.00‰ to − 30.00‰, and floating plant when it is about − 35.50‰ [7].

The CAR in closed basins of the Qilian Mountains since the LGM was calculated by the following equation [2, 38].

1 $$CAR = SAR \times OC\left( {\text{\% }} \right) \times \rho \times \left( {1 - \phi } \right)$$

where SAR represents sediment accumulation rate based on age-depth data from individual lake records, OC is average organic carbon content during different periods, ρ is sediment density calculated by Eq. (2) [38], and φ (%) is porosity calculated by Eq. (3) [4].

2 $$\rho = 2.65 - 0.0523\, \times \,{\text{OC}}\left( \% \right)$$

3 $$\varphi = \left( {1 - \frac{{{\text{DBD}}}}{\rho }} \right) \times 100\%$$

However, many lake records have no dry bulk density (DBD) data, thus for those without measured DBD values we use the following empirical relationships [4, 13].

4 $$DBD = 1.665 \times \left( {OC} \right)^{ - 0.887} {\kern 1pt} \quad \left( {{\text{OC}} > {6}\% } \right)$$

5 $$DBD = 1.776 - 0.363 \times \ln \left( {10 \times OC} \right)\quad ({\text{OC}}\underline{ \le } {6}\% )$$

Finally, lake organic carbon storage (LOCS) was calculated as

6 $$LOCS = A \times t \times CAR$$

where A is lake area, t represents the duration of different time periods.

### Evaluations of organic carbon sink and human impact in extant closed basins

In this study, we compiled 39 lake records containing reliable organic carbon content data in closed basins from published peer-reviewed literature (Fig. 4, Additional file 1: Table S4). These lakes are mostly located in the northern mid-latitudes. In order to reasonably estimate the organic carbon sinks in extant closed-basin lakes under a limited data condition, a reassignment of weight coefficients defined in this study is applied. Lake carbon sinks in closed basins are greatly affected by the geophysical-chemical conditions of lake water. Accordingly, data on lake area, pH, alkalinity, salinity from 82 closed-basin lakes were assembled from published literatures (Additional file 1: Table S5). These lakes covering 63.35% of lake area in global closed basins, were further divided into three different types (carbonate, sulfate and chlorine) using information provided in the original publications. The discriminant analysis results indicate that pH values of the three different type of lakes have distinctive signatures and could be used to calculate organic carbon sinks under equilibrium conditions. As the lake area can directly influence the magnitude of lake organic carbon storage, we calculated the weight coefficient by pH × lake area, to represent the proportion of organic carbon content in the three different lake types in extant closed-basin lakes.

The CAR in 39 closed-basin lakes with available TOC data was calculated using Eq. (1). Then CAR was multiplied by different weight coefficients according to their lake types classified above. Organic carbon storage in extant closed-basin lakes was calculated using Eq. (6), where A is total lake area, t represents deposition time span, and CAR is the mean carbon accumulation rate in extant closed-basin lakes. The modern soil organic carbon storage was calculated as

7 $$SOCS = A \times SOCD$$

where SOCD represents soil organic carbon density derived from the HWSD v1.2 [56], and A is area of closed basin excluding lake area.

We divided long-term variations of lake carbon accumulation into a part that completely represents natural variations and a human-influenced part that includes information on both natural and anthropogenic factors. According to curve fitting analysis of CAR in the natural part, we can obtain potential natural CAR in the human-influence part, namely excluding the human impacts. Then, the ratio of human impacts in last few thousand years (3000 years in this study) was defined as

8 $$R_{H} = {{\left( {CAR_{H} - CAR_{N} } \right)} \mathord{\left/ {\vphantom {{\left( {CAR_{H} - CAR_{N} } \right)} {CAR_{N} }}} \right. \kern-\nulldelimiterspace} {CAR_{N} }}$$

where CAR_H_ is the CAR containing influence from human activities, CAR_N_ is the CAR excluding human impacts, namely under completely natural conditions.

Because change in lake organic carbon sink can be indicative of basin-wide organic carbon storages, we assumed that human activities result in soil organic carbon emission and lake organic carbon sink simultaneously and proportional coefficients between organic carbon storages in soil and lake are consistent throughout the past three thousand years. Therefore, the magnitude of the soil organic carbon emissions caused by human activities can be evaluated as

9 $$SOC_{E} = LOC \times \left( {{{SSR_{M} } \mathord{\left/ {\vphantom {{SSR_{M} } {LSR_{M} }}} \right. \kern-\nulldelimiterspace} {LSR_{M} }}} \right) \times R_{H} - LOC_{H}$$

where LOC is the lake organic carbon sink over the past three thousand years, SSR_M_ represents the sedimentary rate of modern soil organic carbon from data in Lal [23], LSR_M_ represents the sedimentary rate of modern lake organic carbon from compiled lake records, R_H_ is the ratio of human impact from Eq. (8), and LOC_H_ is the human-induced organic carbon increase in the lake sediments calculated by CAR_H_ minus CAR_N_ in Eq. (8).

## Supplementary Information

**Additional file 1.** Additional figures and tables.

## Data Availability

Boundaries of closed basins used in this study are available from the HydroBASINS product developed by the Conservation Science Program of World Wildlife Fund (WWF) at https://www.hydrosheds.org/page/hydrobasins. The NPP data are available from the Numerical Terradynamic Simulation Group of University of Montana at http://www.ntsg.umt.edu/project/modis/mod17.php. The HWSD v1.2 data are available from Oak Ridge National Laboratory Distributed Active Archive Center at http://dx.doi.org/10.3334/ORNLDAAC/1247. The land use and vegetation types data are available from Resource and Environment Data Cloud Platform at http://www.resdc.cn. Data from closed-basin lake records collected or compiled in this study are included in additional file.

## Abbreviations

AMS: Accelerator Mass Spectrometry
C/N: Carbon–Nitrogen Ratio
C: Carbon
CAM: Crassulacean acid metabolism
CAR: Carbon accumulation rate
CAR_H_: Carbon accumulation rate containing human activities
CAR_N_: Carbon accumulation rate under nature conditions
DBD: Dry bulk density
IPCC: Intergovernmental panel on climate change
LGM: Last glacial maximum
LOC: Lake organic carbon sink
LOC_H_: Human-induce organic carbon storage of terminal lakes
LOCS: Lake organic carbon storage
LSR: Sedimentary rate of modern lake organic carbon
LSR_M_: Modern lake organic sedimentary rates
NPP: Net primary production
OC: Organic carbon
OSL: Optically Stimulated Luminescence
Pg: Petagram (= 10^15^ g)
pH: Pondus hydrogenii
R_H_: Ratio of human impacts to organic carbon sinks
SAR: Sediment accumulation rate
SOCD: Soil organic carbon density
SOC_E_: Soil organic carbon emissions caused by human activities
SSR: Sedimentary rate of modern soil organic carbon
SSR_M_: Modern soil organic sedimentary rates
TN: Total nitrogen
TOC: Total organic carbon
