# Supplementary material for: Changes of lake organic carbon sinks from closed basins since the Last Glacial Maximum and quantitative evaluation of human impacts
Source: Carbon Balance Manag. 2021 Sep 17;16:28. doi: 10.1186/s13021-021-00191-6 (PMC8447695; doi:10.1186/s13021-021-00191-6)
Supplement: Supplementary file 1 — Additional file 1. Additional figures and tables. [file 13021_2021_191_MOESM1_ESM.doc]

Additional file 1: Table S1 Age data from terminal lake records in closed basins of the Qilian Mountains.

| Depth (cm) | Dating materials | ^14^C age / OSL age  (yr BP) | Calibrated age (cal yr BP) | Laboratory number/Reference |
| --- | --- | --- | --- | --- |
| **Zhuyeze Lake** |  |  |  |  |
| 225 | Organic matter | 1550±60 | 1433 (1316-1551) | LUG96-44 |
| 250 | Organic matter | 2470±90 | 2545 (2351-2740) | LUG96-45 |
| 262 | Shells | 3140±40 | 3355 (3263-3448) | BA05223 |
| 290 | Organic matter | 3300±90 | 3588 (3356-3821) | LUG96-46 |
| 315 | Organic matter | 4130±110 | 4625 (4298-4953) | LUG96-47 |
| 315 | Shells | 4160±40 | 4701 (4571-4831) | BA05224 |
| 360 | Organic matter | 4530±80 | 5165 (4881-5449) | LUG96-48 |
| 425 | Organic matter | 5960±65 | 6802 (6652-6953) | LUG96-49 |
| 425 | Shells | 5920±40 | 6756 (6658-6854) | BA05225 |
| 425 | Pollen concentrates | 6510±40 | 7429 (7322-7494) | BA101234 |
| 537 | Organic matter | 8412±62 | 9411 (9293-9530) | LUG02-25 |
| 561 | Pollen concentrates | 14220±50 | 17299 (16989-17599) | BA101237 |
| 572 | Organic matter | 9183±60 | 10368 (10234-10502) | LUG02-23 |
| **Zhuyeze Lake** |  |  |  |  |
| 250 | Quartz grain (OSL) | 2270±190 | / | Long et al., 2010 |
| 290 | Quartz grain (OSL) | 3650±300 | / | Long et al., 2010 |
| 375 | Quartz grain (OSL) | 5310±600 | / | Long et al., 2010 |
| 455 | Quartz grain (OSL) | 7180±630 | / | Long et al., 2010 |
| 495 | Quartz grain (OSL) | 7650±670 | / | Long et al., 2010 |
| 560 | Quartz grain (OSL) | 9720±810 | / | Long et al., 2010 |
| **Sanjiiaocheng** |  |  |  |  |
| 25.00 | / | 6990±130 | / | Zhang et al., 2004; Shi et al., 2002 |
| 85.31 | / | 2430±50 | 2492 (2353-2548) | Zhang et al., 2004; Shi et al., 2002 |
| 113.48 | / | 3050±95 | 3239 (2973-3450) | Zhang et al., 2004; Shi et al., 2002 |
| 125.96 | / | 3660±95 | 3994 (3702-4248) | Zhang et al., 2004; Shi et al., 2002 |
| 141.05 | / | 3930±60 | 4363 (4225-4524) | Zhang et al., 2004; Shi et al., 2002 |
| 253.32 | / | 6400±75 | 7332 (7172-7434) | Zhang et al., 2004; Shi et al., 2002 |
| 311.27 | / | 7860±135 | 8712 (8406-9017) | Zhang et al., 2004; Shi et al., 2002 |
| 352.52 | / | 8140±140 | 9083 (8682-9432) | Zhang et al., 2004; Shi et al., 2002 |
| 451.71 | / | 10700±260 | 12551 (11772-13100) | Zhang et al., 2004; Shi et al., 2002 |
| 538.43 | / | 14790±250 | 17997 (17411-18591) | Zhang et al., 2004; Shi et al., 2002 |
| 583.90 | / | 15750±450 | 19063 (18003-20107) | Zhang et al., 2004; Shi et al., 2002 |
| **Yanchi Lake** |  |  |  |  |
| 22.5 | Bulk organic matter | 1850±100 | 1781(1541-1996) | LUG01-05 |
| 47.5 | Pollen concentrates | 6775±40 | 7626(7576-7676) | BA101261 |
| 67.5 | Bulk organic matter | 6415±90 | 7339(7164-7493) | LUG01-06 |
| 87.5 | Pollen concentrates | 13030±50 | 15701(15168-16349) | BA101263 |
| 107.5 | Pollen concentrates | 17250±60 | 20453(20195-21025) | BA101264 |
| 132.5 | Pollen concentrates | 16340±60 | 19486(19382-19833) | BA101265 |
| 147.5 | Pollen concentrates | 17440±70 | 20770(20394-21213) | BA101266 |
| 162.5 | Pollen concentrates | 14830±60 | 18057(17721-18510) | BA101267 |
| 222.5 | Pollen concentrates | 16780±70 | 19949(19576-20246) | BA101268 |
| 247.5 | Pollen concentrates | 21310±90 | 25453(25063-25864) | BA101269 |
| 277.5 | Pollen concentrates | 17830±70 | 21323(20966-21545) | BA101270 |
| 282.5 | Pollen concentrates | 15290±60 | 18581(18156-18718) | BA101271 |
| 317.5 | Pollen concentrates | 10290±40 | 12069(11833-12378) | BA101272 |
| 407.5 | Pollen concentrates | 14390±60 | 17505(17170-17840) | BA101275 |
| **Juyanze Lake** |  |  |  |  |
| 34 | Organic matter | 1786±22 | 1692(-72/+48) | Hartmann & Wünnemann, 2009 |
| 263 | Organic matter | 3127±29 | 3355(-85/+35) | Hartmann & Wünnemann, 2009 |
| 404 | Carbonate of KIA16851 | 6495±35 | 7425(-95/+7) | Hartmann & Wünnemann, 2009 |
| 404 | Organic matter | 4605±28 | 5391(-91/+59) | Hartmann & Wünnemann, 2009 |
| 523 | Organic matter | 5190±30 | 5930(-13/+57) | Hartmann & Wünnemann, 2009 |
| 724 | Organic matter | 7013±41 | 7873(-83/+57) | Hartmann & Wünnemann, 2009  Hartmann & Wünnemann, 2009 |
|  | *Humic acid* | 6530±40 |  |  |
| 820 | Organic matter | 9416±114 | 10772(-322/+378) | Hartmann & Wünnemann, 2009 |
| **Huahai Lake** |  |  |  |  |
| 38 | Bulk organic matter | 290±70 | 324(145-503) | Wang et al., 2013 |
| 73 | Bulk organic matter | 1890±80 | 1808(1614-2001) | Wang et al., 2013 |
| 75 | Bulk organic matter | 7290±165 | 5476(5049-5903) | Wang et al., 2013 |
| 308 | Grass seed | 10660±40 | 9133(9010-9255) | Wang et al., 2013 |
| 308 | Grass seed | 10520±45 | 8871(8720-9021 | Wang et al., 2013 |
| 308 | Grass seed | 10530±70 | 8866(8641-9091) | Wang et al., 2013 |
| 373 | Grass seed | 11800±60 | 10470(10282-10658) | Wang et al., 2013 |
| 665 | Grass | 11900±340 | 10691(9673-11708) | Wang et al., 2013 |
| 673 | Grass seed | 11910±40 | 10633(10523-10743) | Wang et al., 2013 |
| 680 | Grass seed | 12200±160 | 11091(10578-11604) | Wang et al., 2013 |
| 683 | Grass | 11940±160 | 10732(10294-11170) | Wang et al., 2013 |
| 735 | Grass | 12730±155 | 11963(11326-12600) | Wang et al., 2013 |
| 863 | Bulk organic matter | 13080±55 | 12592(12396-12788) | Wang et al., 2013 |
| 925 | Bulk organic matter | 13740±120 | 13074(12952-13196) | Wang et al., 2013 |
| 925 | Terrestrial plant (branch) | 11181±54 | 13074(12952-13196) | Wang et al., 2013 |
| 975 | Bulk organic matter | 13970±55 | 13323(13221-13425) | Wang et al., 2013 |
| 1044 | Bulk organic matter | 14105±55 | 13462(13310-13614) | Wang et al., 2013 |
| 1044 | Bulk organic matter | 14380±250 | 13847(13205-14489) | Wang et al., 2013 |
| **Yitang Lake** |  |  |  |  |
| 145 | Quartz grain (OSL) | 2080±270 | / | Zhao et al.,2015 |
| 745 | Quartz grain (OSL) | 9040±97 | / | Zhao et al.,2015 |
| 1610 | Quartz grain (OSL) | 10940±1190 | / | Zhao et al.,2015 |
| 2150 | Quartz grain (OSL) | 23380±2420 | / | Zhao et al.,2015 |
| **Gahai Lake** |  |  |  |  |
| 1.15 | Inorganic matter | 4100±35 | 3902 | Guo, 2012 |
| 57.38 | Inorganic matter | 7530±40 | 7229 | Guo, 2012 |
| 115.9 | Inorganic matter | 6370±35 | 6823 | Guo, 2012 |
| 172.13 | Inorganic matter | 5530±40 | 5779 | Guo, 2012 |
| 230.66 | Inorganic matter | 5545±40 | 5409 | Guo, 2012 |
| 288.15 | Inorganic matter | 5940±40 | 5997 | Guo, 2012 |
| 348.22 | Inorganic matter | 6995±35 | 6806 | Guo, 2012 |
| 398.11 | Inorganic matter | 6020±40 | 6469 | Guo, 2012 |
| 450.04 | Inorganic matter | 7155±35 | 7072 | Guo, 2012 |
| 500.95 | Inorganic matter | 7010±40 | 6911 | Guo, 2012 |
| 551.85 | Inorganic matter | 9235±45 | 9107 | Guo, 2012 |
| 608.52 | Inorganic matter | 11080±50 | 10954 | Guo, 2012 |
| 656.06 | Inorganic matter | 11610±50 | 11161 | Guo, 2012 |
| 706.57 | Inorganic matter | 9140±50 | 9606 | Guo, 2012 |
| 757.11 | Inorganic matter | 9270±40 | 9488 | Guo, 2012 |
| 803.61 | Inorganic matter | 9415±45 | 9442 | Guo, 2012 |
| 845.36 | Inorganic matter | 9345±40 | 9794 | Guo, 2012 |
| 900.08 | Inorganic matter | 10460±50 | 10500 | Guo, 2012 |
| 952.64 | Inorganic matter | 9815±40 | 9932 | Guo, 2012 |
| 1000.92 | Inorganic matter | 18680±80 | 18000 | Guo, 2012 |
| 1058.85 | Inorganic matter | 13285±50 | 12925 | Guo, 2012 |
| 1125.56 | Inorganic matter | 14870±55 | 14357 | Guo, 2012 |
| 1175.56 | Inorganic matter | 13575±50 | 13209 | Guo, 2012 |
| 1224.44 | Inorganic matter | 20200±90 | 19079 | Guo, 2012 |
| 1286.67 | Inorganic matter | 16450±65 | 15590 | Guo, 2012 |
| 1342.22 | Inorganic matter | 19040±110 | 17739 | Guo, 2012 |
| 1400 | Inorganic matter | 16985±70 | 16625 | Guo, 2012 |
| **Donggi Cona Lake** |  |  |  |  |
| 0 | Total organic carbon | 2290±35 | / | Opitz et al., 2012 |
| 76 | Total organic carbon | 5154±35 | 2868 | Opitz et al., 2012 |
| 116 | Total organic carbon | 6470±50 | 4570 | Opitz et al., 2012 |
| 210 | Total organic carbon | 10560±70 | 9032 | Opitz et al., 2012 |
| 241 | Total organic carbon | 12600±60 | 11829 | Opitz et al., 2012 |
| 271 | Total organic carbon | 13600±80 | 12973 | Opitz et al., 2012 |
| 400 | Total organic carbon | 13090±70 | 12574 | Opitz et al., 2012 |
| 475 | Total organic carbon | 16540±110 | 16955 | Opitz et al., 2012 |
| 572 | Total organic carbon | 18480±200 | 18851 | Opitz et al., 2012 |
| **Hala Lake** |  |  |  |  |
| 53 | Bulk organic matter | 1740±30 | 1639±77 | Liu, 2014 |
| 110 | Bulk organic matter | 2610±35 | 2741±40 | Liu, 2014 |
| 169 | Bulk organic matter | 3995±35 | 4473±62 | Liu, 2014 |
| 221 | Bulk organic matter | 5155±35 | 5915±49 | Liu, 2014 |
| 263 | Bulk organic matter | 6730±40 | 7605±50 | Liu, 2014 |
| **Qinghai Lake** |  |  |  |  |
| 120 | / | 2400±100 | 2357 | Shen et al., 2004 |
| 230 | / | 5060±90 | 5863 | Shen et al., 2004 |
| 355 | / | 6760±180 | 7598 | Shen et al., 2004 |
| 475 | / | 9660±140 | 11151 | Shen et al., 2004 |
| 675 | / | 14820±180 | 17733 | Shen et al., 2004 |
| 745 | / | 15610±90 | 18642 | Shen et al., 2004 |
| **Chaka Salt lake** |  |  |  |  |
| 447-453 | Total organic carbon | 4395±30 | 2802(2754-2851) | Liu et al., 2008 |
| 508-511 | Total organic carbon | 5070±35 | 3624(3554-3694) | Liu et al., 2008 |
| 546-548 | Total organic carbon | 5705±45 | 4493(4401-4586) | Liu et al., 2008 |
| 577-579 | Total organic carbon | 6815±35 | 5789(5749-5830) | Liu et al., 2008 |
| 642-643 | Total organic carbon | 9035±50 | 8114(8021-8208) | Liu et al., 2008 |
| 695-696 | Total organic carbon | 11740±65 | 11547(11270-11824) | Liu et al., 2008 |
| 705-706 | Total organic carbon | 11840±50 | 11820(11601-12041) | Liu et al., 2008 |
| 749-750 | Total organic carbon | 12995±85 | 13159(12997-13321) | Liu et al., 2008 |
| 798-799 | Total organic carbon | 12054±65 | 12190(11979-12190) | Liu et al., 2008 |
| 867-868 | Total organic carbon | 10900±120 | 10437(10168-10707) | Liu et al., 2008 |
| **Genggahai Lake** |  |  |  |  |
| 0-1.16 | Plant residue | 1010±35 | 53(33-73) | Song et al., 2012 |
| 35.86-37.02 | Plant residue | 1125±35 | 141(11-271) | Song et al., 2012 |
| 106.42-107.58 | Plant residue | 2915±35 | 1831(1736-1926) | Song et al., 2012 |
| 107.58-108.74 | Seed | 2545±35 | 1436(1352-1520) | Song et al., 2012 |
| 200.12-201.28 | Plant residue | 3545±35 | 2618(2489-2748) | Song et al., 2012 |
| 235.98-237.14 | Plant residue | 4255±35 | 3475(3391-3558) | Song et al., 2012 |
| 273.00-273.97 | Plant residue | 3740±35 | 2839(2758-2920) | Song et al., 2012 |
| 325.23-326.2 | Plant residue | 4975±35 | 4436(4348-4523) | Song et al., 2012 |
| 391.97-392.93 | Plant residue | 5700±35 | 5448(5319-5577) | Song et al., 2012 |
| 483.68-484.80 | Seed | 5655±35 | 5255(5060-5450) | Song et al., 2012 |
| 497.15-498.28 | Plant residue | 5820±35 | 5537(5470-5604) | Song et al., 2012 |
| 569.01-570.13 | Seed | 5975±35 | 5727(5604-5844) | Song et al., 2012 |
| 572.37-573.50 | Plant residue | 5100±35 | 4628(4444-4812) | Song et al., 2012 |
| 572.37-573.50 | Seed | 5540±35 | 5181(5050-5311) | Song et al., 2012 |
| 574.62-575.74 | Seed | 5520±60 | 5143(4965-5320) | Song et al., 2012 |
| 597.07-598.20 | Plant residue | 8890±35 | 8684(8587-8780) | Song et al., 2012 |
| 633.00-634.13 | Plant residue | 4735±35 | 4099(3974-4233) | Song et al., 2012 |
| 657.83-658.96 | Plant residue | 10300±40 | 10478(10370-10568) | Song et al., 2012 |
| 701.86-702.98 | Plant residue | 12365±40 | 13220(13136-13304) | Song et al., 2012 |
| 780.87-782.00 | Plant residue | 14995±50 | 16655(16282-17028) | Song et al., 2012 |

Additional file 1: Table S2 Site information of terminal lakes in closed basins of the Qilian Mountains.

| **Lake names** | **Corresponding basins** | **Latitude (℃)** | **longitude (℃)** | **Elevation (m)** | **Proxies** | **References** |
| --- | --- | --- | --- | --- | --- | --- |
| Zhuyeze Lake | The Shiyang River drainage basin | 39.05 | 103.67 | 1309 | TOC, C/N, δ^13^C | This Paper |
| Sanjiaocheng | The Shiyang River drainage basin | 38.16 | 102.95 | 1325 | TOC, δ^13^C | Zhang et al., 2004 |
| Yanchi Lake | The Heihe River drainage basin | 39.73 | 99.27 | 1300-1600 | TOC, C/N | This Paper |
| Juyanze Lake | The Heihe River drainage basin | 41.86 | 101.75 | 892-907 | TOC | Hartmann & Wünnemann, 2009 |
| Huahai Lake | The Shule River drainage basin | 40.42 | 97.75 | 1195-1250 | TOC, C/N | Wang et al., 2013 |
| Yitang Lake | The Shule River drainage basin | 40.52 | 94.97 | 1042 | TOC, C/N | Zhao et al., 2015 |
| Gahai Lake | The Qaidam drainage basin | 37.13 | 97.55 | 2850 | TOC, C/N, δ^13^C | Guo, 2012 |
| Donggi Cona Lake | The Qaidam drainage basin | 35.30 | 97.53 | 4090 | TOC, C/N | Opitz et al., 2012 |
| Hala Lake | The Qinghai Lake drainage basin | 38.22 | 97.38 | 4100 | TOC | Liu, 2014 |
| Qinghai Lake | The Qinghai Lake drainage basin | 37.53 | 99.60 | 4583 | TOC, C/N, δ^13^C | Shen et al., 2005 |
| Chaka Salt Lake | The Qinghai Lake drainage basin | 36.68 | 99.12 | 3200 | TOC | Liu et al., 2008 |
| Genggahai Lake | The Qinghai Lake drainage basin | 36.18 | 100.10 | 3000 | TOC, C/N, δ^13^C | Song et al., 2012 |

Additional file 1: Table S3 Organic carbon accumulation rates in closed-basin lakes of the Qilian Mountains during the modern, mid-Holocene and LGM periods.

| **Lake names** | **Modern OC (%)** | **Modern CAR (g C m^-2^ year^-1^)** | **Mid-Holocene OC (%)** | **Mid-Holocene CAR (g C m^-2^ year^-1^)** | **LGM OC (%)** | **LGM CAR (g C m^-2^ year^-1^)** |
| --- | --- | --- | --- | --- | --- | --- |
| Huahai Lake | 0.61 | 3.90 | 0.69 | 2.45 | / | / |
| Sanjiaocheng | / | / | 3.51 | 6.18 | 2.00 | 2.81 |
| Zhuyeze Lake | 0.27 | 8.53 | 0.30 | 5.07 | / | / |
| Juyanze Lake | / | / | 0.74 | 11.88 | / | / |
| Donggi Cona Lake | / | / | 1.65 | 2.67 | 0.26 | 1.70 |
| Genggahai Lake | 2.06 | 39.46 | 3.44 | 8.37 | / | / |
| Chaka Salt Lake | 0.06 | 1.98 | 0.50 | 1.92 | 0.42 | 1.71 |
| Hala Lake | 4.47 | 10.81 | 4.94 | 7.72 | / | / |
| Qinghai Lake | 2.88 | 7.89 | 4.72 | 6.03 | 0.32 | 1.72 |
| Gahai Lake | 2.04 | 13.12 | 1.57 | 7.49 | / | / |
| Yitang Lake | / | / | 0.30 | 4.55 | 0.15 | 1.41 |
| Yanchi Lake | 0.60 | 0.85 | 0.60 | 0.56 | / | / |

Additional file 1: Table S4 Site information of terminal lakes in global closed basins.

| **Lake names** | **Latitude (℃)** | **longitude (℃)** | **Elevations** | **Proxies** | **References** |
| --- | --- | --- | --- | --- | --- |
| Ulaan Nuur | 44.53 | 103.63 | 1110 | TOC, C/N | Lee et al., 2013 |
| Huahai Lake | 40.42 | 97.75 | 1195-1250 | TOC, C/N | Wang et al., 2013 |
| Sanjiaocheng | 38.16 | 102.95 | 1325 | TOC, δ^13^C | Zhang et al., 2004 |
| Zhuyeze Lake | 39.05 | 103.67 | 1309 | TOC, C/N, δ^13^C | This Paper |
| Hoton Nuur | 48.67 | 88.30 | 2083 | TOC, C/N, δ^13^C | Rudaya and Li, 2013 |
| Bogoria Lake | 0.25 | 36.10 | 989 | TOC | Cort et al., 2013 |
| Juyanze Lake | 41.88 | 101.85 | 892-907 | TOC | Hartmann & Wünnemann, 2009 |
| Bear lake | 41.93 | 111.33 | 1805 | TOC | Dean et al., 2006 |
| Abiyata Lake | 7.67 | 38.75 | 1550-1700 | TOC | Legesse et al., 2002 |
| Chamo Lake | 5.85 | 37.65 | 1235 | TOC | Kassa, 2015 |
| Zigetang Co | 32.07 | 90.83 | 4560 | TOC, C/N, δ^13^C | Wu et al., 2007 |
| Zabuye Salt Lake | 38.30 | 97.58 | 4421 | TOC | Wang et al., 2002 |
| Donggi Cona Lake | 35.30 | 98.53 | 4090 | TOC, C/N | Opitz et al., 2012 |
| Nam Co | 30.70 | 90.67 | 4718 | TOC | Zhu et al., 2008 |
| Garba Guracha Lake | 6.83 | 39.75 | 400-4200 | TOC | Tiercelin et al., 2008 |
| Karaqul Lake | 39.02 | 73.53 | 3915 | TOC, C/N | Heinecke et al., 2017 |
| Jenny Lake | 43.76 | -110.73 | 2070 | TOC, C/N, δ^13^C | Larsen et al., 2016 |
| Yoa Lake | 19.05 | 21.52 | 380 | TOC | Eggermont et al., 2008 |
| Dali Nor Lake | 42.83 | 116.72 | 1100-1400 | TOC, C/N | Wang et al., 2008 |
| Cuoe Lake | 31.47 | 91.05 | 4532 | TOC, C/N, δ^13^C | Wu et al., 2006 |
| Red rock Lake | 44.62 | -111.83 | 2015 | TOC | Mumma et al., 2012 |
| Genggahai Lake | 36.18 | 100.10 | 3000 | TOC, C/N, δ^13^C | Song et al., 2012 |
| Pyramid Lake | 40.00 | -119.58 | 4500 | TOC | Benson et al., 2002 |
| Chaka Salt Lake | 36.68 | 99.12 | 3200 | TOC | Liu et al., 2008 |
| Hala Lake | 38.30 | 97.58 | 4100 | TOC | Wünnemann et al., 2012 |
| Qinghai Lake | 36.53 | 99.60 | 4583 | TOC, C/N, δ^13^C | Shen et al., 2005 |
| Titicaca Lake | -15.75 | -69.42 | 3810 | TOC | Fritz et al., 2007 |
| Luobupo | 40.78 | 90.05 | 780 | TOC, C/N, δ^13^C | Luo et al., 2008 |
| Gahai Lake | 37.13 | 97.55 | 2850 | TOC, C/N, δ^13^C | Guo, 2012 |
| Tengger Nuur | 42.45 | 110.70 | 1092 | TOC | Guo et al., 2012 |
| Yanhaizi Lake | 40.13 | 108.45 | 1180 | TOC, C/N | Chen et al., 2003 |
| Manito Lake | 52.75 | -109.72 | 600 | TOC | Kuhry et al., 1992 |
| Aibi Lake | 44.85 | 82.38 | 194 | TOC | Wu, 1995 |
| Yitang Lake | 40.52 | 94.97 | 1042 | TOC, C/N | Zhao et al., 2015 |
| Balikun Lake | 43.67 | 92.80 | 1580 | TOC | Xue & Zhang, 2011 |
| Yanchi Lake | 39.73 | 99.27 | 1300-1600 | TOC, C/N | This paper |
| Bosten Lake | 42.08 | 87.05 | 1048 | TOC, C/N | Zhang et al., 2007 |
| Balkhash Lak | 40.25 | 91.10 | 330 | TOC | Feng et al., 2013 |
| Redberry Lake | 52.72 | -109.15 | 532 | TOC | Stempvoort et al., 1993 |
| Malawi Lake | -10.27 | 34.32 | 457 | C/N, δ^13^C | Castañeda et al., 2009 |
| Lake Son Kul | 41.85 | 75.13 | 3016 | C/N | Huang et al., 2014 |
| Bonneville Lake | 40.50 | -112.25 | 1555 | δ^13^C | Oviatt et al., 2003 |
| Lake Elsinore | 33.66 | -117.35 | 900 | δ^13^C | Kirby et al., 2010 |

Additional file 1: Table S5 Terminal lakes of 82 closed basins around the world with their data on surface area, pH, alkalinity, and salinity.

| **Lake names** | **Location** | **Latitude (℃)** | **longitude (℃)** | **Basin Types** | **Lake surface area (km2)** | **Catchment area (km2)** | **PH** | **Alkalinity (μ eq/L)** | **Salinity (g/L)** | **References** |
| --- | --- | --- | --- | --- | --- | --- | --- | --- | --- | --- |
| Red Pond | [America](../../../../../lenovo/AppData/Local/youdao/DictBeta/Application/7.5.0.0/resultui/dict/%3Fkeyword=America) | 34.85 | -109.43 | Carbonate | 0.0016 | / | 8.9 | 552000 | 33 | Duarte et al., 2008; Cole et al., 1967 |
| Green Pond | [America](../../../../../lenovo/AppData/Local/youdao/DictBeta/Application/7.5.0.0/resultui/dict/%3Fkeyword=America) | 34.85 | -109.43 | Carbonate | 0.0011 | / | 8.9 | 808000 | 33 | Duarte et al., 2008; Cole et al., 1967 |
| Big Soda Lake | [America](../../../../../lenovo/AppData/Local/youdao/DictBeta/Application/7.5.0.0/resultui/dict/%3Fkeyword=America) | 39.52 | -118.87 | Carbonate | 1.62 |  | 9.9 | 48000 | 3.8 | Kimmel et al., 1978 |
| Soap Lake | [America](../../../../../lenovo/AppData/Local/youdao/DictBeta/Application/7.5.0.0/resultui/dict/%3Fkeyword=America) | 47.41 | -119.50 | Carbonate | 5.2 |  | 9.8 | / | 15 | Galat et al., 1981; Edwards, 2015 |
| Abiyata Lake | Ethyopia | 7.67 | 38.75 | Carbonate | 205 |  | 9.55 | 246400 | 9.5 | Tudorancea & Zullini, 1989 |
| Shala Lake | Ethyopia | 7.50 | 38.50 | Carbonate | 307.79 | 4156.2 | 9.5 | 316200 | 14.3 | Tudorancea & Zullini, 1989 |
| Langano Lake | Ethyopia | 7.61 | 38.80 | Carbonate | 229.28 | 2007.6 | 9 | 12200 | 1.1 | Tudorancea & Zullini, 1989 |
| Abaya Lake | Ethyopia | 6.28 | 37.95 | Carbonate | 1084.75 | 16336 | 8.7 | 8600 | 0.6 | Tudorancea & Zullini, 1989 |
| Chamo Lake | Ethyopia | 5.85 | 37.65 | Carbonate | 551 |  | 9.2 | 13300 | 1 | Tudorancea & Zullini, 1989 |
| Aranguadi Lake | Ethyopia | 8.75 | 38.98 | Carbonate | 0.54 | / | 10.3 | 51400 | 4.4 | Tudorancea & Zullini, 1989 |
| Kilotes Lake | Ethyopia | 8.75 | 38.98 | Carbonate | 0.77 | / | 9.6 | 63400 | 4.4 | Tudorancea & Zullini, 1989 |
| Chitu Lake | Ethyopia | 7.50 | 38.50 | Carbonate | 0.8 | / | 10 | 239000 | 44.9 | Tudorancea & Zullini, 1989 |
| Zwai Lake | Ethyopia | 7.98 | 38.86 | Carbonate | 411.96 | 7296.3 | 9.1 | / | / | Tudorancea & Zullini, 1989 |
| Awassa Lake | Ethyopia | 7.05 | 38.46 | Carbonate | 86.8 | 1457.6 | 9 | / | / | Tudorancea & Zullini, 1989 |
| Pawlo Lake | Ethyopia | 8.75 | 38.98 | Carbonate | 0.5 | / | 9.2 | 10000 | / | Green, 1986 |
| Bishoftu Lake | Ethyopia | 8.75 | 38.98 | Carbonate | 0.3 | / | 9.2 | 20000 | / | Green, 1986 |
| Garba Guracha Lake | Ethyopia | 6.86 | 39.85 | Carbonate | 0.15 | 3 | 6.77 |  |  | Tiercelin et al., 2008 |
| Emeentatita Lake | Kenya | -0.45 | 36.25 | Carbonate | 18.77 | 470.1 | 9.5 | 19672 | 2.9 | Mwaura, 1999 |
| Baringo Lake | Kenya | 0.60 | 36.07 | Carbonate | 125.43 | 6604.4 | 8.6 | 10100 | 0.8 | Oduor et al., 2003 |
| Sonachi Lake | Kenya | -0.78 | 36.27 | Carbonate | 0.18 | 1 | 9.6 | 105000 | 6.6 | Ballot et al., 2005 |
| Simbi Lake | Kenya | -0.37 | 34.63 | Carbonate | 0.29 | / | 10.55 | 260000 | 12.2 | Ballot et al., 2005 |
| Bogoria Lake | Kenya | 0.25 | 36.10 | Carbonate | 36.25 | 760.7 | 10.2 | 1500000 | 49 | Ballot et al., 2004 |
| Nakuru Lake | Kenya | 0.33 | 36.25 | Carbonate | 40 | 800 | 10.5 | 122000 | 6.8 | Ballot et al., 2004 |
| Elmenteita Lake | Kenya | 0.45 | 36.25 | Carbonate | 20 | 500 | 9.85 | 400000 | 25.8 | Mwirichia et al., 2011 |
| Chad Lake | Chad | 13.07 | 14.53 | Carbonate | 1875.52 | 980211.9 | 8.6 | 12180 | 1.7 | Duarte et al., 2008 |
| Nam Co | China | 30.70 | 90.67 | Carbonate | 1963.82 | 10741.3 | 9.13 | / | 1.8 | Zhu et al., 2008 |
| Zigetang Co | China | 32.07 | 90.83 | Carbonate | 191.4 | 3430 | 10 | / | 41 | Wu et al., 2007; Lei et al., 2010 |
| Baisha | China | 40.76 | 114.90 | Carbonate | 0.65 | / | 9 | 6800 | 0.98 | Zhao & He, 1999 |
| Shuiquan | China | 40.76 | 114.90 | Carbonate | 4.5 | / | 9.1 | 15400 | 1.87 | Zhao & He, 1999 |
| Cuoe Lake | China | 31.47 | 91.05 | Carbonate | 61.3 | 1081 | 9.8 | / | 12.06 | Wu et al., 2006 |
| Donggi Cona Lake | China | 35.30 | 98.53 | Carbonate | 231.17 | 3157.5 | 8.85 | / | / | Opitz et al., 2012 |
| Zabuye Salt Lake | China | 38.30 | 97.58 | Carbonate | 242 | 6680 | 9.7 | / | 200-460 | Wang et al., 2002 |
| Hoton Nuur | [Mongolia](https://mapcarta.com/Mongolia) | 48.67 | 88.30 | Carbonate | 50.1 | / | 7.5 | / | / | Rudaya et al., 2008 |
| Lake Van | Turkey | 38.63 | 49.82 | Carbonate | 3568.73 | 17045.8 | 9.8 | 155000 | / | Reimer et al., 2009 |
| Mariout Lake | Egypt | 31.15 | 29.88 | Carbonate | 50 | / | 9.35 | 6000 | 4.8 | Duarte et al., 2008 |
| Chappice Lake | Canada | 50.17 | -110.37 | Sulfate | 1.5-2.2 | / | 8.3975 | 117750 | / | Birks & Remeda, 1999 |
| Redberry Lake | Canada | 52.72 | -109.15 | Sulfate | 45 | / | / | / | 23 | Duarte et al., 2008; Galat et al., 1981 |
| Manito Lake | Canada | 52.75 | -109.72 | Sulfate | 114 | / | 9.6 | 75400 | 17 | Duarte et al., 2008; Galat et al., 1981 |
| Gorbea Lake | Chile | -25.42 | -68.58 | Sulfate | 27 | / | 7.57 | 1090 | 33 | Risacher et al., 2002 |
| Gahai Lake | China | 37.13 | 97.55 | Sulfate | 35 | / | 7.86 | / | 71.13 | Tang et al., 2018 |
| Aibi Lake | China | 44.85 | 82.38 | Sulfate | 564.86 | 51701.6 | 8.7 | / | 40.92 | Wu, 1995 |
| Balikun Lake | China | 43.67 | 92.80 | Sulfate | 121.34 | 1260.5 | 6.78 | 14850 | / | Xue & Zhang, 2011; Cai and Li, 1994 |
| Yanchi Lake | China | 39.73 | 99.27 | Sulfate | 150 | 6000 | 8.25 | / | / | Li et al., 2013 |
| Agmon Lake | Israel | 33.10 | 35.60 | Sulfate | 1.1 | / | 7-8.5 | 3100 | / | Markel et al., 1998 |
| Canapa Lake | Bolivia | -21.00 | -68.00 | Sulfate | 1.5 | 227 | 9.18 | 2150 | 11.4 | Williams et al., 1995 |
| Humbolt Lake | [America](../../../../../lenovo/AppData/Local/youdao/DictBeta/Application/7.5.0.0/resultui/dict/%3Fkeyword=America) | 40.00 | -108.62 | Sulfate | 4.4 | / | 8.7 | 4500 | 2.7 | Galat et al., 1981 |
| Balkhash Lake | Kazakhstan | 46.50 | 75.00 | Sulfate | 16717.89 | 404800.5 | 8.85 | 5000 | 2.4 | Duarte et al., 2008; Fairbridge, 1968 |
| Aral Sea | Central Asia | 46.25 | 60.30 | Sulfate | 23865.91 | 622506.9 | 8.31375 | 29333 | 14.9 | Zavialov, 2012; |
| Pyramid Lake | [America](../../../../../lenovo/AppData/Local/youdao/DictBeta/Application/7.5.0.0/resultui/dict/%3Fkeyword=America) | 40.00 | -119.58 | Chlorine | 476.87 | 7162.4 | 9.2 | 28600 | 5.7 | Galat et al., 1981 |
| Mono Lake | [America](../../../../../lenovo/AppData/Local/youdao/DictBeta/Application/7.5.0.0/resultui/dict/%3Fkeyword=America) | 38.00 | -120.00 | Chlorine | 185.96 | 1810.2 | 10 | / | 80-90 | Galat et al., 1981 |
| Abert Lake | [America](../../../../../lenovo/AppData/Local/youdao/DictBeta/Application/7.5.0.0/resultui/dict/%3Fkeyword=America) | 42.58 | -120.25 | Chlorine | 174.35 | 2319 | 10 | 120000 | 33 | Conte and Conte, 1988 |
| Great Salt Lake | [America](../../../../../lenovo/AppData/Local/youdao/DictBeta/Application/7.5.0.0/resultui/dict/%3Fkeyword=America) | 41.00 | -112.50 | Chlorine | 6478.85 | 86895.5 | 8.4 | 131000 | 33 | Duarte et al., 2008; Naftz et al., 2008 |
| Corangamite Lake | America | 38.17 | -143.40 | Chlorine | 2.3 | / | 9.2 | 18800 | 29.2 | Duarte et al., 2008; Galat et al., 1981 |
| Werowrap Lake | America | 38.23 | -143.50 | Chlorine | 0.2 | / | 9.8 | 20800 | 35.4 | Duarte et al., 2008; Galat et al., 1981 |
| Red Rock Lake | America | 44.62 | -111.83 | Chlorine | 10 | 23.33 | 9.7 | 18400 | 23.1 | Duarte et al., 2008; Galat et al., 1981 |
| Ramaditas Lake | Bolivia | -21.63 | -68.08 | Chlorine | 4 | 277 | 7.95 | 2750 | 21 | Williams et al., 1995 |
| Uyuni Lake | Bolivia | -19.00 | -67.67 | Chlorine | 1203.27 | 24943.5 | 7.7 | 2900 | 15 | Sylvestre et al., 2001 |
| Poopo Lake | Bolivia | -18.66 | -67.17 | Chlorine | 2247.91 | 102962.8 | 8.5 | 4400 | 37.1 | Sylvestre et al., 2001 |
| Pastos Grandes Lake | Bolivia | -21.62 | -68.06 | Chlorine | / | 100 | 8.2 | 5700 | 39 | Risacher & Eugster, 2010 |
| Hedionda Lake | Bolivia | -21.57 | -68.05 | Chlorine | 4.4 | 52 | 8.5 | 10000 | 67 | Williams et al., 1995 |
| Colorada Lake | Bolivia | -22.33 | -67.42 | Chlorine | 52 | 875 | 8.4 | 31500 | 120 | Williams et al., 1995 |
| Titicaca Lake | [Bolivia](https://en.wikipedia.org/wiki/Bolivia) | -15.75 | -69.42 | Chlorine | 8002.51 | 56588.1 | 8.6 | 2190 | 1 | Duarte et al., 2008; Bengtsson, 2012 |
| Qinghai Lake | China | 36.53 | 99.60 | Chlorine | 4266.55 | 29604.7 | 9.2 | 30100 | 9 | Duarte et al., 2008; Shen et al., 2004 |
| Chaka Salt Lake | China | 36.68 | 99.12 | Chlorine | 105 | 11600 | 7.6 | / | 341.86 | Tang et al., 2018 |
| Genggahai Lake | China | 36.18 | 100.10 | Chlorine | 2 | / | 9.1 | / | / | Song et al., 2012 |
| Hala Lake | China | 38.30 | 97.58 | Chlorine | 585.6 | 4737.5 | 8.8 | / | 15.4 | Wünnemann et al., 2012 |
| Huanggai | China | 40.76 | 114.90 | Chlorine | 3.8 | / | 8.6 | 11900 | 1.93 | Zhao & He, 1999 |
| Erquanjing Ⅰ | China | 40.76 | 114.90 | Chlorine | 0.02 | / | 8.5 | 9500 | 1.94 | Zhao & He, 1999 |
| Danmu | China | 40.76 | 114.90 | Chlorine | 0.53 | / | 8.3 | 9070 | 2.96 | Zhao & He, 1999 |
| Gonghui | China | 40.76 | 114.90 | Chlorine | 0.53 | / | 8.9 | 15600 | 3.34 | Zhao & He, 1999 |
| Xiaohongshi | China | 40.76 | 114.90 | Chlorine | 0.7 | / | 8.8 | 27900 | 6 | Zhao & He, 1999 |
| Duikou | China | 40.76 | 114.90 | Chlorine | 2.02 | / | 9.4 | 22600 | 8 | Zhao & He, 1999 |
| Erquanjing Ⅱ | China | 40.76 | 114.90 | Chlorine | 0.02 | / | 9.3 | 27600 | 8.3 | Zhao & He, 1999 |
| Kulun | China | 40.76 | 114.90 | Chlorine | 5.34 | / | 9.6 | 66100 | 10.5 | Zhao & He, 1999 |
| Shitou | China | 40.76 | 114.90 | Chlorine | 0.08 | / | 9.6 | 48900 | 43.8 | Zhao & He, 1999 |
| Wubaiqing | China | 40.76 | 114.90 | Chlorine | 1.67 | / | 8.8 | 25700 | 49.8 | Zhao & He, 1999 |
| Erquanjing Ⅲ | China | 40.76 | 114.90 | Chlorine | 0.02 | / | 8.3 | 39300 | 175.2 | Zhao & He, 1999 |
| Dead Sea | Israel | 31.50 | 35.50 | Chlorine | 643.18 | 43271.9 | 6.2 | 3400 | 342 | Duarte et al., 2008; Vestal, 2008 |
| Wadi El Natrun | Egypt | 30.25 | 30.50 | Chlorine | 272 |  | 9.1 | 7612 | 330 | Duarte et al., 2008; Taher, 1999 |
| Urmia Lake | Iran | 37.50 | 46.00 | Chlorine | 4932.76 | 52200.3 | 7.866667 | 8190 | 138.3 | Eimanifar & Mohebbi, 2007 |
| Caspian sea | Central Asia | 42.00 | 50.50 | Chlorine | 377001.91 | 1404108 | 8.05 | 3370 | 12.5 | Duarte et al., 2008; Mora et al., 2004 |
| Issyk-Kul | Kyrgyzstan | 42.40 | 77.20 | Chlorine | 6195.93 | 21917 | 8.7 | 5700 | 5.7 | Ricketts et al., 2001 |

Additional file 1: Table S6 Terminal lakes of closed basins with their organic carbon accumulation rates during the modern, mid-Holocene and LGM periods.

| **Lake names** | **Basins** | **Modern OC (%)** | **Modern CAR (g C m^-2^ year^-1^)** | **Mid-Holocene OC (%)** | **Mid-Holocene CAR (g C m^-2^ year^-1^)** | **LGM OC (%)** | **LGM CAR (g C m^-2^ year^-1^)** |
| --- | --- | --- | --- | --- | --- | --- | --- |
| Ulaan Nuur | Carbonate | 0.55 | 0.47 | 2.99 | 0.64 | 0.36 | 2.53 |
| Huahai Lake | Carbonate | 0.61 | 0.57 | 3.90 | 0.65 | 0.35 | 2.45 |
| Sanjiaocheng | Carbonate | / | / | / | 3.29 | 0.04 | 6.18 |
| Zhuyeze Lake | Carbonate | 0.27 | 2.24 | 8.53 | 0.95 | 0.81 | 5.07 |
| Hoton Nuur | Carbonate | 1.25 | 0.04 | 0.41 | 1.77 | 0.19 | 3.13 |
| Bogoria Lake | Carbonate | 3.69 | 1.50 | 43.73 | / | / | / |
| Juyanze Lake | Carbonate | / | / | / | 0.88 | 1.01 | 11.88 |
| Bear lake | Carbonate | 2.01 | 0.74 | 10.26 | 1.14 | 0.21 | 2.15 |
| Abijata Lake | Carbonate | 3.24 | 4.22 | 70.21 | / | / | / |
| Chamo Lake | Carbonate | 1.22 | 5.65 | 59.76 | 1.93 | 0.81 | 11.13 |
| Zigetang Co | Carbonate | 1.48 | 1.10 | 13.01 | 1.75 | 0.63 | 8.03 |
| Zabuye Salt Lake | Carbonate | 0.02 | 0.59 | 0.32 | 0.36 | 0.59 | 2.42 |
| Donggi Cona Lake | Carbonate | / | / | / | 1.63 | 0.63 | 2.67 |
| Nam Co | Carbonate | 0.52 | 4.71 | 28.70 | 0.97 | 0.11 | 0.99 |
| Garba Guracha Lake | Carbonate | 10.88 | 1.11 | 24.23 | 12.07 | 0.70 | 15.16 |
| Karaqul Lake | Carbonate | 3.06 | 0.53 | 8.20 | 3.12 | 0.41 | 6.40 |
| Jenny Lake | Carbonate | 1.75 | 0.09 | 1.10 | 2.48 | 0.16 | 2.33 |
| Yoa Lake | Carbonate | 2.34 | 1.50 | 20.23 | 5.32 | 1.02 | 17.28 |
| Cuoe Lake | Carbonate | 1.64 | 0.56 | 7.04 | 3.10 | 0.47 | 7.18 |
| Dali Nor Lake | Carbonate | / | / | / | 6.91 | 0.35 | 5.42 |
| Red rock Lake | Chlorine | 18.41 | 0.14 | 3.20 | 23.44 | 0.39 | 8.97 |
| Genggahai Lake | Chlorine | 2.06 | 2.83 | 39.46 | 3.50 | 0.10 | 8.37 |
| Pyramid Lake | Chlorine | 5.78 | 2.96 | 51.95 | / | / | / |
| Chaka Salt Lake | Chlorine | 0.06 | 1.62 | 1.98 | 0.57 | 0.27 | 1.92 |
| Hala Lake | Chlorine | 4.47 | 0.61 | 10.81 | 4.94 | 0.45 | 7.72 |
| Qinghai Lake | Chlorine | 2.88 | 0.51 | 7.89 | 4.85 | 0.34 | 6.03 |
| Titicaca Lake | Chlorine | / | / | / | 6.63 | 0.24 | 5.94 |
| Luobupo | Sulfate | / | / | / | / | / | / |
| Gahai Lake | Sulfate | 2.04 | 0.94 | 13.12 | 1.57 | 0.62 | 7.49 |
| Tengger Nuur | Sulfate | 1.94 | 0.34 | 4.61 | 0.62 | 0.45 | 3.76 |
| Yanhaizi Lake | Sulfate | 0.25 | 2.06 | 7.53 | 0.25 | 1.38 | 4.77 |
| Manito Lake | Sulfate | 43.53 | 0.82 | 20.91 | 19.84 | 0.33 | 7.53 |
| Aibi Lake | Sulfate | / | / | / | 0.36 | 0.44 | 1.96 |
| Yitang Lake | Sulfate | / | / | / | 0.29 | 1.52 | 4.55 |
| Balikun Lake | Sulfate | 2.60 | 0.29 | 4.54 | 1.51 | 0.29 | 3.35 |
| Yanchi Lake | Sulfate | 0.60 | 0.13 | 0.85 | 0.62 | 0.08 | 0.56 |
| Bosten Lake | Sulfate | 4.72 | 0.70 | 12.45 | 3.76 | 1.15 | 19.19 |
| Balkhash Lak | Sulfate | 1.62 | 2.08 | 25.79 | / | / | / |
| Redberry Lake | Sulfate | 14.39 | 0.68 | 15.30 | / | / | / |

Additional file 1: Table S7 Organic carbon data in closed-basin lakes of the Qilian Mountains and extant closed-basin lakes.

| **Period** | **Closed-basin lakes of Qilian Mountains** | | | |  | **Extant closed-basin lakes** | | | |
| --- | --- | --- | --- | --- | --- | --- | --- | --- | --- |
|  | 22-0 ka | 22-18 ka | 8-4 ka | 3-0 ka |  | 22-0 ka | 22-18 ka | 8-4 ka | 3-0 ka |
| OC (%） | 1.68 | 0.48 | 3.39 | 2.43 |  | 3.27 | 1.51 | 5.69 | 5.10 |
| CAR (g C m^-2^ year^-1^) | 5.52 | 1.76 | 6.31 | 12.97 |  | 4.98 | 2.25 | 6.36 | 10.86 |
| Total carbon burial (Pg) | 0.90 | 0.07 | 0.19 | 0.29 |  | 80.56 | 8.27 | 18.71 | 23.96 |

**
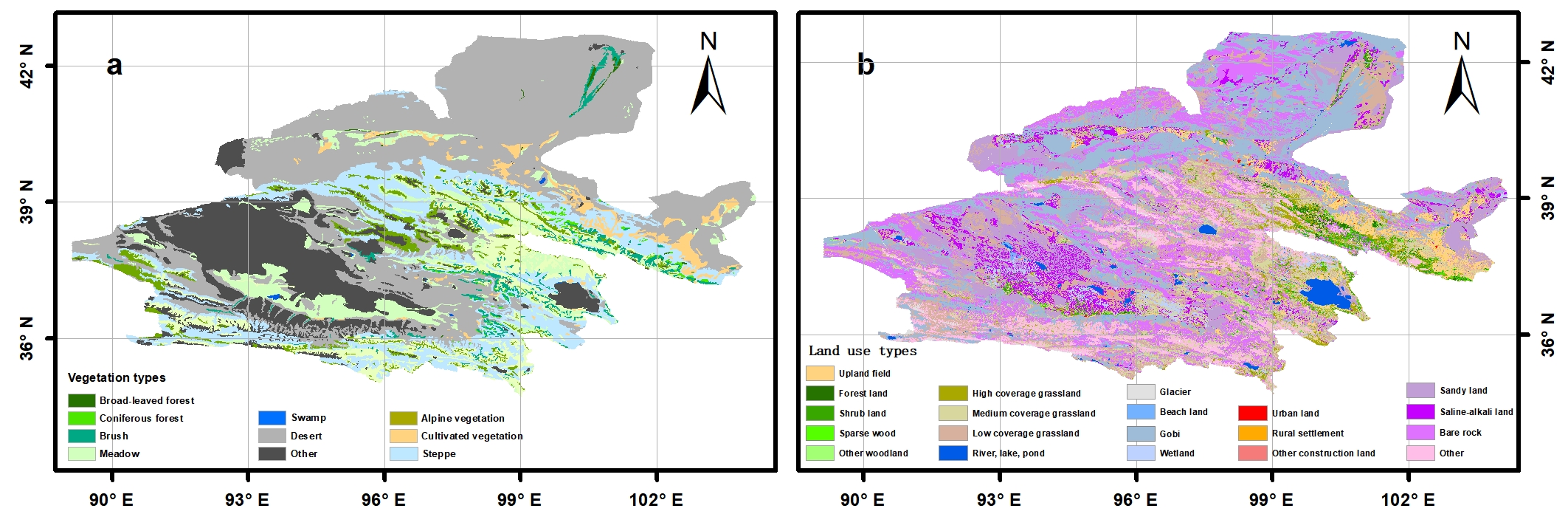
**

**Additional file 1: Fig S1.** Spatial distribution of vegetation (a) and land use (b) types in closed basins of the Qilian Mountains. The data are download from the Resource and Environment Data Cloud Platform.


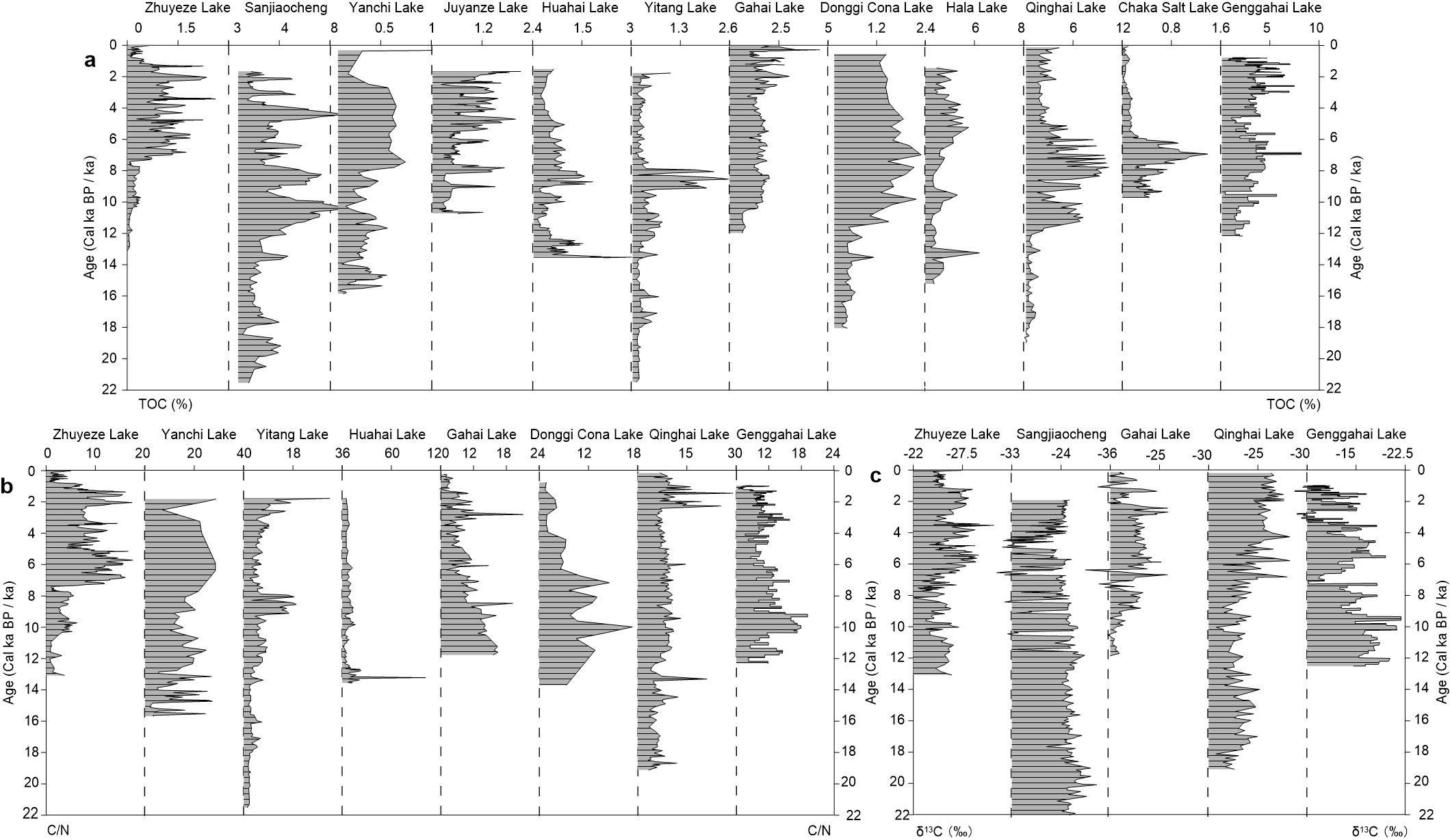


**Additional file 1: Fig S2.** Temporal variation patterns for organic geochemical proxies of terminal lakes in closed basins of the Qilian Mountain (references see Additional file 1: Table S1).

**
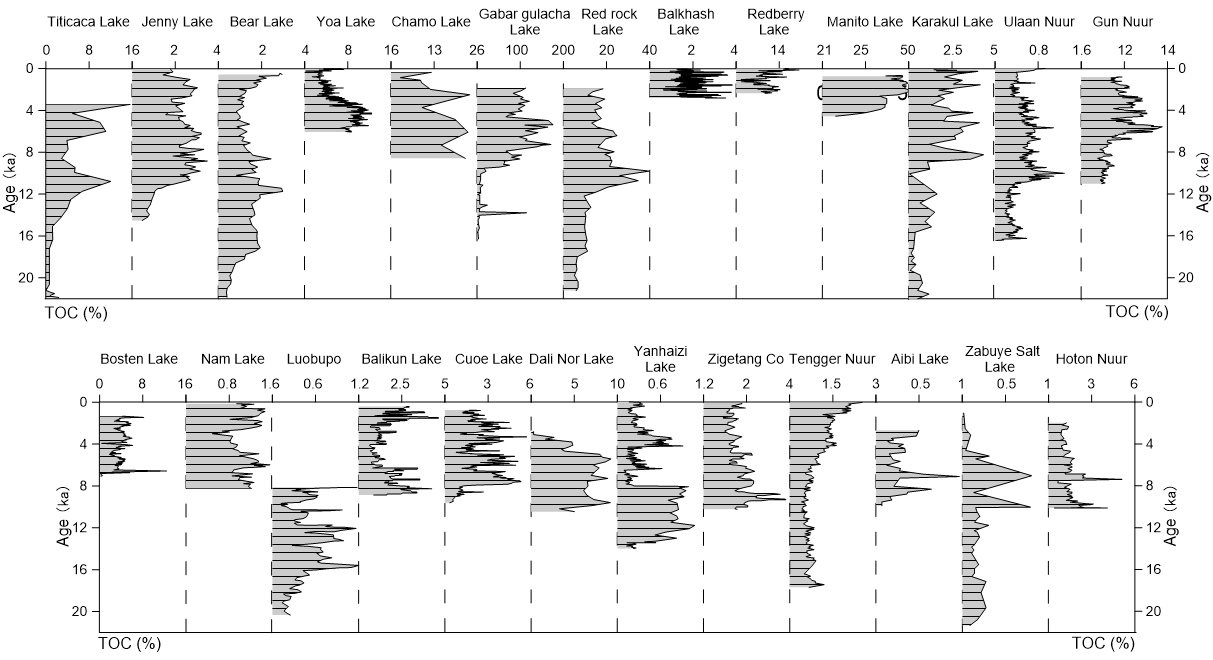
**

**Additional file 1: Fig S3.** Temporal variation patterns for total organic carbon content in extant closed-basin lakes (references see Additional file 1: Table S3).

**
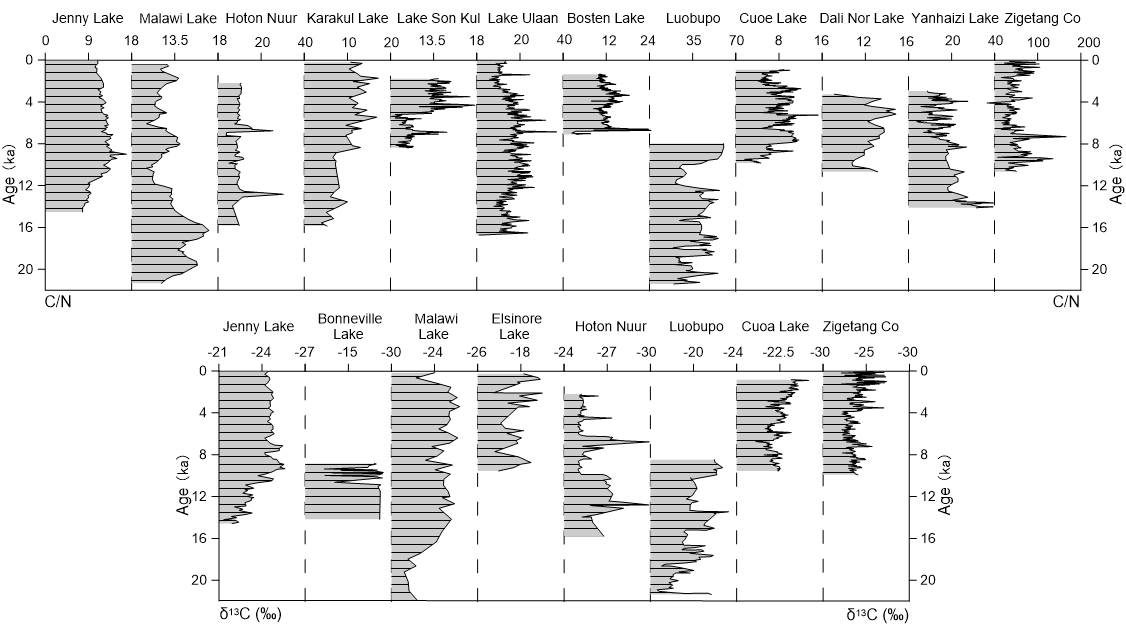
**

**Additional file 1: Fig S4.** Temporal variation patterns for C/N and δ^13^C in extant closed-basin lakes (references see Additional file 1: Table S3).

**
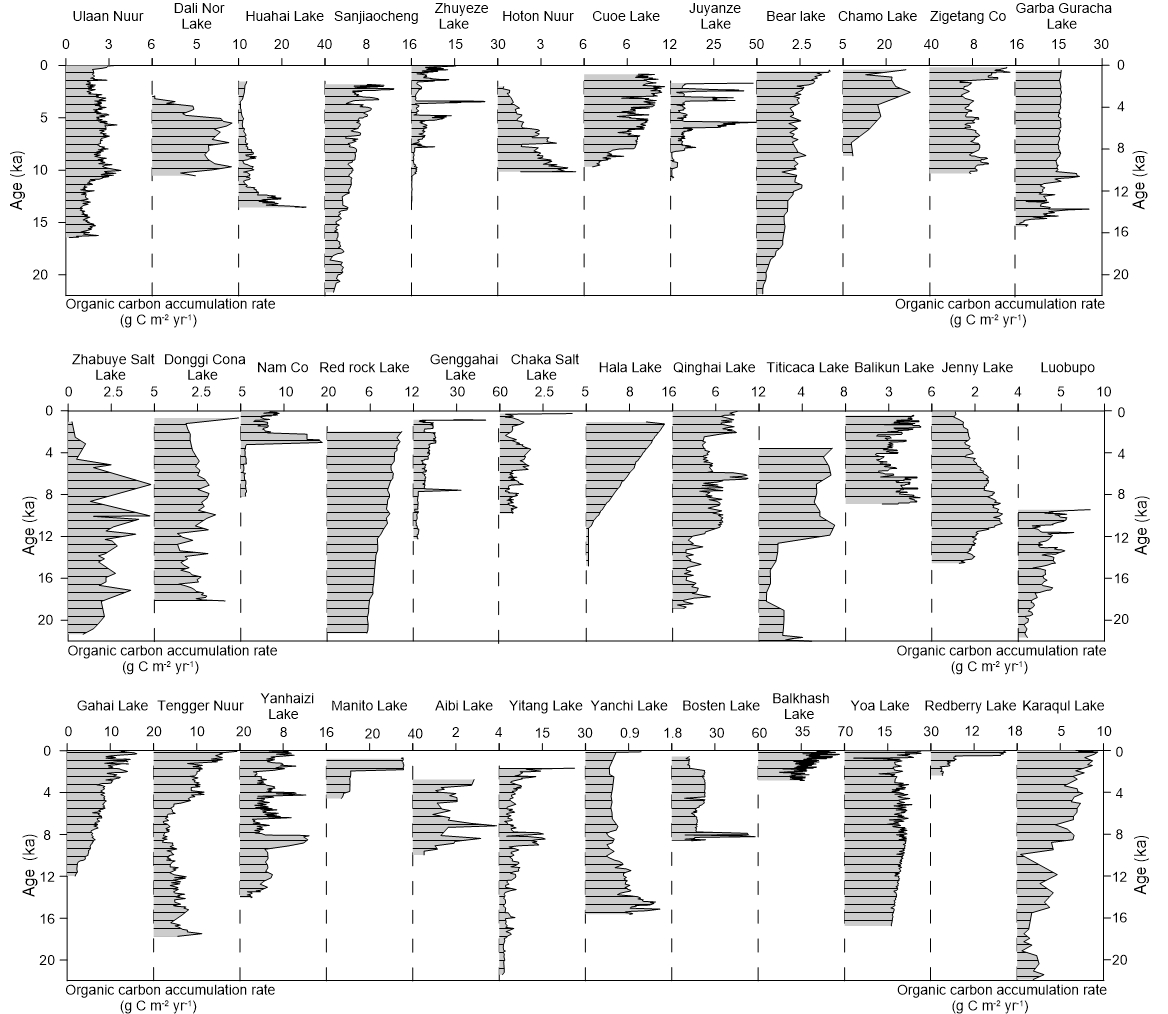
**

**Additional file 1: Fig S5.** Temporal variation patterns for organic carbon accumulation rates in extant closed-basin lakes.

References

Ballot, A., 2004. Cyanobacteria and cyanobacterial toxins in three alkaline rift valley lakes of Kenya - lakes Bogoria, Nakuru, and Elmenteita. J. Plankton. Res. 26, 925-935. <https://doi.org/10.1093/plankt/fbh084.>

Ballot, A., Krienitz, L., Kotut, K., Wiegand, C., Pflugmacher, S., 2005. Cyanobacteria and cyanobacterial toxins in the alkaline crater lakes Sonachi and Simbi, Kenya. Harmful Algae 4, 139-150. <https://doi.org/10.1016/j.hal.2004.01.001.>

Bengtsson, L., 2012. Titicaca lake. Springerplus 275 - 275. https://doi.org/10.1007/978-1-4020-4410-6_102.

Benson, L., Kashgarian, M., Rye, R., Lund, S., Susan, L., 2002. Holocene multidecadal and multicentennial droughts affecting northern California and Nevada. [Quaternary Sci. Rev.](http://www.baidu.com/link?url=rvF4LRB6r8JtK9gdum3_kJZ2izcLXgiuKTUKlrOtIA11qR45ZieVVGFZd6nnEVUUgvm2ggCxXoZtW4p10zQxkdD3GIBw2JrflyHrPAueY5_) 21, 659-682. [https://](https://www.nature.com/articles/348711a0.)doi.org/10.1016/S0277-3791(01)00048-8.

Birks, S.J., Remeda, V.N., 1999. Hydrogeological investigation of Chappice lake, southeastern Alberta: groundwater inputs to a saline basin. J. Paleolimnol. 21, 235-255. https://doi.org/10.1023/A:1008041810022.

Castañeda, I.S., Werne, J.P., Johnson, T.C., Filley, T.R., 2009. Late quaternary vegetation history of southeast africa: the molecular isotopic record from lake Malawi. Palaeogeogra. Palaeoecol. 275, 100-112. [https://](https://www.nature.com/articles/348711a0.)doi.org/10.1016/j.palaeo.2009.02.008.

Chen, C.T.A., Lan, H.C., Lou, J.Y., Chen, Y.C., 2003. The dry Holocene megathermal in Inner Mongolia. Palaeogeogra. Palaeoecol. 193, 181-200. [https://](https://www.nature.com/articles/348711a0.)doi.org/10.1016/s0031-0182(03)00225-6.

Cai, X., Li, X.H., 1994. A study on the hydrochemical of Barcol Salt Lake in Xinjiang and evaluation of Artemia barkolica survival environment. Journal of August 1st agriculture college. 2, 15-20.

Cole, G.A., Brown, W., 1967. Unusual monomixis in two saline arizona ponds. Limnol. Oceanogr. 12, 584-591. https://doi.org/10.2307/2833502.

Conte, F.P., Conte, P.A. 1988. Abundance and spatial distribution of artemia salina in lake Abert, Oregon. Hydrobiologia 158, 167-172. https://doi.org/10.1007/BF00026274.

Cort, G. Bessems, I., Keppens, E., Mees, F., Cumming, B., Verschuren, D., 2013. Late-Holocene and recent hydroclimatic variability in the central Kenya rift valley: the sediment record of hypersaline lakes Bogoria, Nakuru and Elementeita. Palaeogeogra. Palaeoecol*.* 388, 69-80. [https://](https://www.nature.com/articles/348711a0.)doi.org/10.1016/j.palaeo.2013.07.029.

Dean, W., Rosenbaum, J., Skipp, G., Colman, S., Forester, R., Liu, A., Simmons, K., Bischoff, J., 2006. Unusual Holocene and late Pleistocene carbonate sedimentation in Bear lake, Utah and Idaho, USA. Sedimentary Geol. 185, 93-112. [https://](https://www.nature.com/articles/348711a0.)doi.org/10.1016/j.sedgeo.2005.11.016.

Duarte, C.M., Prairie, Y., Montes, C., Cole, J., Striegl, R., Melack, J., Downing, J., 2008. CO2 emissions from saline lakes: a global estimate of a surprisingly large flux. J. Geophys. Res. 113, 10654-10658. https://doi.org/10.1029/2007JG000637.

Edwards, T.C., 2015. PH dependent antibiotic resistance of an alkaliphilic, halotolerant bacterium isolated from Soap lake. (Washington Dissertations).

Eimanifar, A., Feridon, M., 2007. Urmia lake northwest Iran.: a brief review. Saline Systems. 3, 5. https://doi.org/10.1186/1746-1448-3-5.

Eggermont, H., Verschuren, D., Fagot, M., Rumes, B., Bocxlaer, B.V., 2008. Aquatic community response in a groundwater-fed desert lake to Holocene desiccation of the Sahara. [Quaternary Sci. Rev.](http://www.baidu.com/link?url=P6mwURmxArfAx9mtb0VkBZGu9IVx8gCVabOwLGsvzfUaBkFKsPVgLtEiVjzv-PP3hVAdkoUHH4fKGe4PuGCW5SelHu9Kq6SVVX5De6gVSlO) 27, 2411-2425. [https://](https://www.nature.com/articles/348711a0.)doi.org/10.1016/j.quascirev.2008.08.028.

Fairbridge, R.W., 1968. Lake Balkhash. In: Geomorphology. Encyclopedia of Earth Science. Springer, Berlin, Heidelberg. https://doi.org/10.1007/3-540-31060-6_207.

Feng, Z., Wu, H., Zhang, C., Ran, M., Sun, A., 2013. Bioclimatic change of the past 2500 years within the Balkhash basin, eastern Kazakhstan, central Asia. [Quatern. Int.](http://www.baidu.com/link?url=wVlbJiin_GcIJ-aC7a8W2VsI_uIlSnmSe52x0tgWqXXowSssoOQGsgTWLBxV5C_x_3T1P4k5Nq69cIAOUBsu1K) 311, 63-70. <https://doi.org/10.1016/j.quaint.2013.06.032>.

Fritz, S.C., Baker, P.A., Seltzer, G.O., Ballantyne, A., Tapia, P., 2007. Quaternary glaciation and hydrologic variation in the south american tropics as reconstructed from the lake titicaca drilling project. [Quaternary Res](http://www.baidu.com/link?url=dKd8S_oTadwolWTf6HbNrz7c59Tp31s6lSGO7t02_8ViTp1kCeJhh-F89DR5l_Mtm03iKcPr1rgX_qPW6gf1xr63Cmum6KrMfCdszj3Ixie)., 68, 410-420. <https://doi.org/10.1016/j.yqres.2007.07.008>.

Galat, D.L., Lider, E.L., Vigg, S., Robertson, S.R., 1981. Limnology of a large, deep, north American terminal lake, Pyramid lake, Nevada, USA. Hydrobiologia 81-82, 281-317. <https://doi.org/10.1007/978-94-009-8665-7_22.>

Green J., 1986. Zooplankton associations in some Ethiopian crater lakes. Freshwater Biology 16, 495-499. https://doi.org/10.1111/j.1365-2427.1986.tb00992.x.

Guo, J., Li, J., Zhao, Q., Fang, R., Zhang, C. 2012. The grain size and element record of the dry Mid-holocene lake in the Tengger Nuur, Inner Mongolia plateau, China. Marine Geology & Quaternary Geology 32, 115-122. https://doi.org/10.3724/SP. J.1140.2012.05.115.

Guo, X.Y. 2012. Holocene Climate Change Documented by Lake Sediments from Lake Gahai in the Monsoonal Margin Region, Northwest China (Doctoral dissertation).

Heinecke, L., Mischke, S., Adler, K., Barth, A., Biskaborn, B.K., Plessen, B. Nitze, I., Kuhn, G., Rajabov, I., Herzschuh, U., 2017. Climatic and limnological changes at lake karakul Tajikistan. during the last ~29 cal ka. [J. Paleolimnol](http://www.baidu.com/link?url=G12l1Kiox6bAYAVcXhlQ241Vru-B3TbXYSHObMJQbE_jgB7VKcvhLHnXEY7LkfILYvfvqUDJEdxamjlvlBwVKK). 58, 317-334. https://doi.org/10.1007/s10933-017-9980-0.

Huang, X.T., Oberhänsli, H., Mathis, M., Prasad, S., Von Suchodoletz, H., 2012. Hydrological changes in western Central Asia Kyrgyzstan. during the Holocene as inferred from a palaeolimnological study in lake Son Kul. [Quaternary Sci. Rev](http://www.baidu.com/link?url=rvF4LRB6r8JtK9gdum3_kJZ2izcLXgiuKTUKlrOtIA11qR45ZieVVGFZd6nnEVUUgvm2ggCxXoZtW4p10zQxkdD3GIBw2JrflyHrPAueY5_). 103, 134-152. <https://doi.org/10.1016/j.quascirev.2014.09.012>.

Hartmann, K., Wünnemann, B., 2009. Hydrological changes and Holocene climate variations in NW China, inferred from lake sediments of Juyanze palaeolake by factor analyses. [Quatern. Int](http://www.baidu.com/link?url=TkYlUgTVgyI4w-Vso7ZzElLahsnDbvLUJFqrD4O7UCP6uCNM15iBS8-ZYgv-YOdcVih90C2eByUzL0UTaepUhK). 194, 28-44. https://doi.org/10.1016/j. quaint. 2007.06.037.

Kassa, G.T., 2015. Holocene environmental history of lake Chamo, south Ethiopia. (Dissertation).

Kimmel, B.L. Gersberg, R.M., Paulson, L.J., Axler, R.P., Goldman, C.R., 1978. Recent changes in the meromictic status of Big Soda lake, Nevada. Limnol. Oceanogr. 23, 1021-1025. https://doi.org/10.4319/lo.1978.23.5.1021.

Kirby, M.E., Lund, S., Patterson, W., Anderson, M., Bird, B., Ivanovici, B., Ivanovici, L., Monarrez, P., Nielsen, S., 2010. A Holocene record of pacific decadal oscillation pdo.-related hydrologic variability in southern california (lake Elsinore, CA). [J. Paleolimnol](http://www.baidu.com/link?url=eHbwnhyYqMX78OjtNS9o_6KQCm8I8PmPtx9jficxVAm30X3PBVDQktAJhPYSF6Soww_5YF709qMLEdtNsFOw9q). 44, 819-839. https://doi.org/10.1007/s10933-010-9454-0.

Kuhry, P., Halsey, L.A., Bayley, S.E., Vitt, D.H., 1992. Peatland development in relation to Holocene climatic change in Manitoba and Saskatchewan Canada. Canadian J. Earth Sci. 29, 1070-1090. https://doi.org/10.1139/e92-086.

Larsen, D.J., Finkenbinder, M.S., Abbott, M.B., Ofstun, A.R., 2016. Deglaciation and postglacial environmental changes in the teton mountain range recorded at Jenny Lake, Grand Teton national park, WY. [Quaternary Sci. Rev](http://www.baidu.com/link?url=P6mwURmxArfAx9mtb0VkBZGu9IVx8gCVabOwLGsvzfUaBkFKsPVgLtEiVjzv-PP3hVAdkoUHH4fKGe4PuGCW5SelHu9Kq6SVVX5De6gVSlO). 138, 62-75. https://doi.org/10.1016/j.quascirev.2016.02.024.

Lee, M.K., Lee, Y.I., Lim, H.S., Lee, J.I., Yoon, H.I., 2013. Late pleistocene-holocene records from lake Ulaan, southern mongolia: implications for east Asian palaeomonsoonal climate changes. J. Quaternary Sci. 28, 370-378. https://doi.org/10.1002/jqs.2626.

Lei, Y., Zhang, H., Li, S., Yang, L., Zhang, W., 2010. Variation of 13C value in authigenic carbonates from Zigetang Co, Tibetan plateau since 1950 AD. J. Lake Sci. 22, 143-150. https://doi.org/10.1007/s11430-007-0113-x.

Legesse, D., Gasse, F., Radakovitch, O., Vallet-Coulomb, C., Bonnefille, R., Verschuren, D.,Gibert, E., Barker, P., 2002. Environmental changes in a tropical lake lake Abiyata, Ethiopia. during recent centuries. Palaeogeogr. Palaeoecol. 187, 233-258. https://doi.org/10.1016/s0031-0182(02)00479-0.

Liu, S., 2014. Lacustrine Sediment Variations Based on Multi--proxy Records and Potential Implications from Hala Lake, NW China. (Doctoral dissertation).

Liu, X., Dong, H., Rech, J.A., Matsumoto, R., Bo, Y., Wang, Y., 2008. Evolution of Chaka Salt Lake in NW China in response to climatic change during the latest Pleistocene–Holocene. [Quaternary Sci. Rev](http://www.baidu.com/link?url=Xj5W3Tgo_ERxFSG84bPWIanRub77qXd4M-83fCHtryJU8GJyGZDRWolbCeheIbYWmMiRVKfm88Qq3U3xjdjYWYmJfV5N2vfMbqFReL9BokK). 27, 867-879. <https://doi.org/10.1016/j.quascirev.2007.12.006.>

Long, H., Lai, Z., Wang, N., Li, Y. 2010. Holocene climate variations from zhuyeze terminal lake records in east Asian monsoon margin in arid northern China. Quaternary Sci., 74, 46-56. https://doi.org/10.1016/j.yqres.2010.03.009.

Luo, C., Peng, Z., Yang, D., Liu, W., He, J., Liu, G., Zhang, P., 2008. Paleoclimate of Lop Nur and the response to global change by geochemical elements multi—analysis. Geochimica 37, 139-148. [https://](https://www.nature.com/articles/348711a0.)doi.org/10.7666/d.y1412618.

Mora, S.D., Mohammad, R.S., Eric, W., Sabine, A., Roberto, C., 2004. An assessment of metal contamination in coastal sediments of the Caspian sea. Mari. Pollut. Bull. 48, 61-77. https://doi.org/10.1016/s0025-326x(03)00285-6.

Mumma, S.A., Whitlock, C., Pierce, K., 2012. A 28,000 year history of vegetation and climate from lower Red Rock Lake, centennial Valley, southwestern Montana, USA. Palaeogeogr. Palaeoecol. 326-328, 30-41. [https://](https://www.nature.com/articles/348711a0.)doi.org/10.1016/j.palaeo.2012.01.036.

Mwaura, F.A., 1999. Spatio-chemical survey of hydrogeothermal springs in Lake Elementaita, Kenya. Intern. J. Salt Lake Res. 8, 127-138. <https://doi.org/10.1016/j.palaeo.2012.01.036.>

Naftz, D., Angeroth, C., Kenney, T., Waddell, B., Darnall, N., Silva, S., Perschon, C., Whiteheade, L., 2008. Anthropogenic influences on the input and biogeochemical cycling of nutrients and mercury in Great Salt Lake, Utah, USA. Appl. Geochem. 23, 1731-1744. https://doi.org/10.1016/j.apgeochem.2008.03.002.

Oduor, S.O., Schagerl, M., Mathooko, J.M., 2003. On the limnology of lake Baringo Kenya.: i.temporal physico-chemical dynamics. Hydrobiologia 506-509, 121-127. https://doi.org/10.1023/b:hydr.0000008563.00000.18.

Markel, D., Sass, E., Lazar, B., Bein, A., 1998. Biogeochemical evolution of a sulfur-iron rich aquatic system in a reflooded wetland environment (lake Agmon, northern Israel). Wetl. Ecol. Manag. 6, 103-120. https://doi.org/10.1023/a:1008407800060.

Mwirichia, R., Cousin, S., Muigai, A.W., Boga, H.I., Stackebrandt, E., 2011. Bacterial diversity in the Haloalkaline Lake Elmenteita, Kenya. Curr. Microbiol. 62, 209-221. https://doi.org/10.1007/s00284-010-9692-4.

Opitz S., Wünnemann B., Aichner B., Dietze E., Hartmann K., Herzschuh U., IJmker J., Lehmkuhl F., Li S., Mischke S., Plotzki A., Stauch G., Diekmann B., 2012. Late glacial and Holocene development of Lake Donggi Cona, north-eastern Tibetan plateau, inferred from sedimentological analysis. Palaeogeogr. Palaeoecol. s337-338, 159-176. [https://](https://www.nature.com/articles/348711a0.)doi.org/10.1016/j.palaeo.2012.04.013.

Oviatt, C.G., Madsen, D.B., Schmitt, D.N., 2003. Late pleistocene and early holocene rivers and wetlands in the bonneville basin of western north america. Quaternary Research Orlando, 60, 200-210. [https://](https://www.nature.com/articles/348711a0.)doi.org/10.1016/s0033-5894(03)00084-x.

Reimer, A., Günter, L., Stephan, K., 2009. Lake van, eastern Anatolia, hydrochemistry and history. Aquat. Geochem. 15, 195-222. https://doi.org/10.1007/s10498-008-9049-9.

Ricketts, R.D., Thomas, C.J., Erik, T.B., Kenneth, A.R., Vladimir, V.R., 2001. The Holocene paleolimnology of lake Issyk-kul, Kyrgyzstan: trace element and stable isotope composition of ostracodes. Palaeogeogr. Palaeoecol. 176, 207-227. <https://doi.org/10.1016/S0031-0182(01)00339-X>.

Risacher, F., Alonso, H., Salazar, C., 2002. Hydrochemistry of two adjacent acid saline lakes in the Andes of northern Chile.  Chem. Geol. 187, 39-57. https://doi.org/10.1016/S0009-2541(02)00021-9.

Risacher, F., Eugster, H.P., 2010. Holocene pisoliths and encrustations associated with spring-fed surface pools, Pastos Grandes, Bolivia. Sedimentology 26, 253-270. https://doi.org/10.1111/j.1365-3091.1979.tb00353.x.

Rudaya, N., Li, H.C., 2013. A new approach for reconstruction of the Holocene climate in the Mongolian Altai: the high-resolution δ^13^C records of TOC and pollen complexes in Hoton-nur lake sediments. J. Asian Earth. Sci. 69, 185-195. https://doi.org/10.1016/j.jseaes.2012.12.002.

Smoak, J.M., Swarzenski, P.W., 2004. Recent increases in sediment and nutrient accumulation in bear lake, Utah/Idaho, USA. Hydrobiologia 525, 175-184.
https://doi.org/10.1023/B:HYDR.0000038865.16732.09.

Shen, J, Liu, X.Q., Ryo, M., Wang, S.M., Yang, X.D., 2004. A high-resolution climatic change since the Late Glacial Age inferred from multi-proxy of sediments in Qinghai Lake. Sci. China Earth Sci. 34, 582-589. https://doi.org/10.1360/03yd0148.

Song, L., Qiang, M.R., Lang, L.L., Wang, Q., Li, M.Z., 2012. Changes in palaeoproductivity of Genggahai Lake over the past 16 ka in the Gonghe Basin, northeastern Qinghai-Tibetan plateau. [Chinese Sci. Bull.](http://www.baidu.com/link?url=FqoU3cCPvlntp7yClE8JAg2BC6mex7bxGNwK-PXDPG4st8-zOKa0KLEfhUGELbx3ET4ysoqu2Yvv0gpcgc1dQa) 57, 2595-2605. https://doi.org/10.1007/s11434-012-5191-2.

Stempvoort, D.R.V., Edwards, T.W.D., Evans, M.S., Last, W.M., 1993. Paleohydrology and paleoclimate records in a saline prairie lake core : mineral, isotope and organic indicators. [J. Paleolimnol](http://www.baidu.com/link?url=eHbwnhyYqMX78OjtNS9o_6KQCm8I8PmPtx9jficxVAm30X3PBVDQktAJhPYSF6Soww_5YF709qMLEdtNsFOw9q). 8, 135-147. https://doi.org/10.1007/bf00119786.

Sylvestre, F., Servant-Vildary, S., Roux, M. 2001. Diatom-based ionic concentration and salinity models from the south Bolivian Altiplano 15–23°S. [J. Paleolimnol](http://www.baidu.com/link?url=xBQ_bX4hrquSOP5PrU3kYPwOBTQ7L4jm6eEoF2mzF1v0j9LE9-wV72NG7NrzkPtQZFQ0m6p1FbeJVaKxBYsUga). 25, 279-295. <https://doi.org/10.1023/A:1011157611619.>

Taher, A.G., 1999. Inland saline lakes of Wadi EI Natrun depression, Egypt.  J. Salt Lake Res. 8, 149-169. https://doi.org/10.1007/BF02442128.

Tang, Y., Yang, Y.C., Yang, J., Jiang, H.C., 2018. Gene diversity involved in Kalvin pathway of carbon fixation and its response to environment variables in surface sediments of the Northern Qinghai-Tibetan Lake. Earth Sci. 43, 23-34. <https://doi.org/10.1007/BF02442128.>

Tiercelin, J., Gibertb, E., Umer, M., Bonnefille, R., Disnar, J., Lézine, A., Hureau-Mazaudier, D., Travi, Y., Keravis, D., Lambi, H., 2008. High-resolution sedimentary record of the last deglaciation from a high-altitude lake in Ethiopia. Quaternary Sci. Rev.  27, 449-467. <https://doi.org/10.1016/j.quascirev.2007.11.002.>

Tudorancea, C., Zullini, A., 1989. Associations and distribution of benthic nematodes in the Ethiopian rift valley lakes. Hydrobiologia 179, 81-96. https://doi.org/10.1007/bf00011932.

Vestal, S., 2008. Bridging the gap between call centers and the Web. Climatic Changes and Water Resources in the Middle East and North Africa. Springer Berlin Heidelberg. https://doi.org/10.1007/978-3-540-85047-2.

Wang, H., Liu, H., Liu, Y., Cui, H., 2008. Mineral magnetism of lacustrine sediments and Holocene palaeoenvironmental changes in dali nor area, southeast inner Mongolia plateau, China. Palaeogeogr. Palaeoclimatol. 208, 175-193. https://doi.org/10.1016/j.palaeo.2004.02.026.

Wang, R.L., Scarpitta, S.C., Zhang, S.C., Zheng, M.P., 2002. Later Pleistocene/Holocene climate conditions of Qinghai–Xizhang plateau Tibet based on carbon and oxygen stable isotopes of Zabuye lake sediments. Earth Planetary Sci. Lett. 203, 461-477. https://doi.org/10.1016/s0012-821x(02)00829-4.

Wang, N., Li, Z., Li, Y., Cheng, H., 2013. Millennial-scale environmental changes in the Asian monsoon margin during the Holocene, implicated by the lake evolution of Huahai lake in the Hexi corridor of northwest China. [Quatern. Int](http://www.baidu.com/link?url=TkYlUgTVgyI4w-Vso7ZzElLahsnDbvLUJFqrD4O7UCP6uCNM15iBS8-ZYgv-YOdcVih90C2eByUzL0UTaepUhK). 313-314, 100-109. http://dx.doi.org/10.1016/j.quaint.2013.08.039.

Williams, W.D., Carrick, T.R., Bayly, I.A.E., Green, J., Herbst, D.B., 1995. Invertebrates in salt lakes of the Bolivian Altiplano. Int. Salt Lake Res. 4, 65-77. http://doi.org/10.1007/BF01992415.

Wu, Y., Lücke, A., Jin, Z., Wang, S., Schleser, G.H., Battarbee, R.W., Xia, W.L., 2006. Holocene climate development on the central tibetan plateau: a sedimentary record from Cuoe lake. Palaeogeoger. Palaoeocl. 234, 328-340. http://doi.org/10.1016/j.palaeo.2005.09.017.

Wu, J., 1995. Characters of the evolution of climate and environment during the last 10 ka years in Aibi Lake basin, Xinjiang. Scientia Geographica Sinica 15, 40-46.

Wu, Y., Wang, S., Hou, X., 2006. Chronology of Holocene lacustrine sediments in Co Ngoin, central Tibetan plateau. Science China Press 49, 991-1001. http://doi.org/10.1007/s11430-006-0991-3.

Wu, Y.H., Lücke, A., Wünnemann, B., Li, S.J., Wang, S.M., 2007. Holocene climate change in the central Tibetan plateau inferred by lacustrine sediment geochemical records. Science China Press 50, 1548-1555. http://doi.org/10.1007/s11430-007-0113-x.

Wünnemann, B., Zhang, Y., Yan, D., Wang, R., Shen, Y., Fang, X., Zhang, J.W., 2012. Implications of diverse sedimentation patterns in Hala lake, Qinghai province, China for reconstructing late Quaternary climate. J. Paleolimnol. 48, 725-749. http://doi.org/10.1007/s10933-012-9641-2.

Xue, J.B., Zhang, W., 2011. Holocene climate variation denoted by Barkol lake sediments in northeastern Xinjiang and its possible linkage to the high and low latitude climates. Sci. China Earth Sci. 54, 603-614. http://doi.org/10.1007/s11430-010-4111-z.

Zavialov, P., 2012. Ongoing changes in physical and chemical regimes of the Aral sea. Quatern. Int. 279-280, 553-554. http://doi.org/10.1016/j.quaint.2012.08.1954.

Zhang, C.J., Chen, F.H., Shang, H.M., Cao, J., 2004. The paleoenvironmental significance of organic carbon isotope in lacustrine sediments in the arid China: an example from Sanjiaocheng palaeolake in Minqin. Quaternary Sci. 24, 88-94. https://doi.org/10.3321/j.issn:1001-7410.2004.01.011.

Zhang, C.J., Zhen, M.P., Prokopenko, A., Steffen, M., Kuang, J., Yang, Q.L., Guo, F.Q., Fen, Z.D., 2007. The palaeoenvironmental variation from the high-resolution record of the Holocene sediment carbonate and isotopic composition in Bosten Lake and responding to glacial activity. Acta Geologica Sinica 81, 1658-1671.

Zhao, L.Y., Lv, H., Zhang, E., Wang, X., Ge, S., Chen, Y., Zhang, H., Wu, B., 2015. Lake-level and paleoenvironment variations in Yitang lake northwestern China. during the past 23ka revealed by stable carbon isotopic composition of organic matter of lacustrine sediments. Quaternary Sci., 35, 172-179. https://doi.org/10.11928/j.issn.1001-7410.2015.01.16.

Zhao, W., He, Z.H., 1999. Biological and ecological features of inland saline waters in North Hebei, China. J. Salt Lake Res. 8, 267-285. https://doi.org/10.1007/bf02449936.

Zhu, L., Wu, Y., Wang, J., Xiao, L., Ju, J., Xie, M., Li, M., Mäusbacher, R., Schwalb, J., Daut, G., 2008. Environmental changes since 8.4 ka reflect in the lacustrine core sediments from Nam Co, central Tibetan Plateau, China. Holocene 18, 831-839. https://doi.org/10.1177/0959683608091801.
